# Unsupervised risk factor identification across cancer types and data modalities via explainable artificial intelligence

**DOI:** 10.1038/s41746-026-02663-w

**Published:** 2026-05-11

**Authors:** Maximilian Ferle, Jonas Ader, Thomas Wiemers, Nora Grieb, Beatrice Berneck, Adrian Lindenmeyer, Hartmut Goldschmidt, Elias K. Mai, Uta Bertsch, Hans-Jonas Meyer, Thomas Neumuth, Markus Kreuz, Kristin Reiche, Maximilian Merz

**Affiliations:** 1 Center for Scalable Data Analytics and Artificial Intelligence (ScaDS.AI) Dresden/Leipzig, Universität Leipzig, Leipzig, Germany; 2 Innovation Center Computer Assisted Surgery (ICCAS), University of Leipzig, Leipzig, Germany; 3 Department of Diagnostics, Fraunhofer Institute for Cell Therapy and Immunology, Leipzig, Germany; 4 Department of Hematology, Hemostaseology, Cellular Therapy and Infectiology, University Hospital of Leipzig, Leipzig, Germany; 5 Department of Internal Medicine V, University Hospital Heidelberg, Heidelberg, Germany; 6 National Center for Tumor Diseases (NCT), Heidelberg, Germany; 7 Department of Diagnostic and Interventional Radiology, University Hospital Leipzig, Leipzig, Germany; 8 Institute for Clinical Immunology, University Hospital of Leipzig, Leipzig, Germany; 9 Myeloma Service, Memorial Sloan Kettering Cancer Center, New York, NY USA

**Keywords:** Cancer, Computational biology and bioinformatics, Oncology

## Abstract

Risk stratification is an important tool in clinical decision-making, yet current approaches often fail to translate sophisticated survival analysis into actionable clinical criteria. We present a novel method for training any neural network architecture on any data modality to identify prognostically distinct patient groups by directly optimizing for survival heterogeneity across patient clusters. We evaluate the method in simulation experiments and demonstrate its utility in practice by applying it to two distinct cancer types: analyzing laboratory parameters from multiple myeloma (MM) patients using the CoMMpass dataset and computed tomography images from non-small cell lung cancer (NSCLC) patients using the Lung1 dataset. Post-hoc explainability analyses uncover clinically meaningful features determining group assignments, which align well with established risk factors in both cases. Our findings in MM were externally validated using the GMMG-MM5 study dataset, while the NSCLC findings were validated with data from our own institution, thus lending strong weight to the method’s utility. This pan-cancer, model-agnostic approach enables the discovery of novel prognostic signatures across diverse data types while providing interpretable results that promise to complement treatment personalization and clinical decision-making in oncology and beyond.

## Introduction

Survival analysis is a vital statistical method in healthcare that examines the time until clinical events such as disease progression or death occur while accounting for variable follow-up periods. Risk stratification, built on these principles, guides treatment decisions and resource allocation across a variety of domains by enabling clinicians to categorize patients into groups of differing prognosis based on prognostic markers^1^. While statistical approaches like Cox proportional hazards models^2^ and random survival forests^3^ enable researchers to quantify the relative impact of variables on survival outcomes, these methods face operational challenges in clinical practice, because they often fall short of providing clear decision boundaries^4,5^ that would facilitate straightforward stratification into risk groups and their interpretation requires expert domain knowledge^6,7^. Due to this limitation, these models find little direct application in day-to-day clinical decision-making^8,9^. One example of clinically useful patient grouping is demonstrated by the R-ISS^10^ for multiple myeloma (MM), which has proven its practical value in oncology care by stratifying patients into three distinct risk groups based on clearly defined boundaries for laboratory parameters and cytogenetic findings. The success of the R-ISS in clinical practice stems from its ability to translate complex prognostic information into clear, actionable decision points that guide treatment selection and intensity^11^. While sophisticated machine learning approaches have revolutionized many aspects of survival analysis^12^, their potential to aid patient grouping based on risk factors remains surprisingly limited in scope. Current methodologies mostly either rely on clustering patients based on feature similarity metrics that may not necessarily correlate with survival patterns^13,14^, using recursive partitioning techniques that offer little insight on an individual patient basis^15^, or modeling survival functions through elaborate mixture models that may not capture the influence of underlying biological predictors^16,17^ or require a priori knowledge of survival times^18^. The challenge of clustering survival data based on patient features has remained largely unaddressed in computational medicine, with existing approaches typically focusing on proxy metrics, but not their direct relationship.

A variety of recent efforts have proposed models, with the main focus primarily lying on optimizing architecture design that implicitly embeds survival objectives for specific data modalities and sometimes dedicated use-cases (e.g. tabular data^16,19,20^, medical imaging^21,22^, or time series^23^ modeling). Although effective within their target area, their requirements for specific data modalities or specific applications, narrows their potential for transferability across different domains. Unlike prior methods that embed survival objectives implicitly within model architecture, our work instead shifts the focus from architecture design to the parameterization of the optimization problem itself. Concretely, we introduce a probabilistic relaxation of the multivariate logrank statistic and derive a differentiable optimization criterion that (1) explicitly ties cluster assignments to survival-discriminative probabilities and (2) offers compatibility with arbitrary neural network architectures as a plug-and-play loss function.

This parameterization-centric approach provides a unified, model-agnostic way to train survival-guided clustering across heterogeneous modalities, as demonstrated by our successful application to both multi-layer perceptrons (MLPs) processing laboratory parameters and convolutional neural networks (CNNs) analyzing computed tomography (CT) images, which identified prognostically distinct patient subgroups with significantly different survival outcomes in both cases.

### Partial multivariate logrank loss

We developed a novel approach to patient stratification by reformulating the multivariate logrank statistic as a differentiable optimization criterion suitable for neural network training. Our implementation leverages PyTorch’s^24^ automatic differentiation capabilities through a custom loss module, which we coined *PartialMultivariateLogRankLoss*. The framework leverages predictions of probability distributions *p*_*i*,*g*_ over possible group assignments, while the classical logrank statistic uses binary indicators *δ*_*g*,*i*_ (refer to methods section *Development of a differentiable optimization criterion*). This generalization enables backpropagation through the computational graph, while maintaining mathematical consistency with the original statistic. The module processes three inputs: predicted group assignment probabilities, survival times, and event indicators to compute the *PartialMultivariateLogRankLoss* as a measure for survival heterogeneity as the optimization criterion. We thereby formalized the core optimization problem of survival-based clustering itself, creating a universal framework that enables any neural network to stratify patients irrespective of data modality and model architecture.

As the logrank is a convex function (imbalanced group assignments maximize the logrank), we augmented the loss function with a penalty term to prevent trivial solutions in the loss calculation:

1 $${L}_{{\rm{total}}}={L}_{{\rm{logrank}}}-\lambda P(p)$$

where *P*(*p*) penalizes imbalanced group assignments with penalty weight *λ*, which can be adjusted through hyperparameter tuning. The penalty function is designed to encourage uniform group sizes through a transformation that maintains differentiability:

2 $$P(p)=\frac{1}{k}\mathop{\sum }\limits_{i=1}^{k}\frac{1}{{p}_{i}^{\alpha }-{({p}_{i}^{\alpha })}^{2}}-4$$

with $\alpha =\frac{ln(1/2)}{ln(1/k)}$ for *k* groups. This formulation creates an asymmetric barrier that bottoms out at 1/*k*, effectively counteracting the tendency of the logrank loss to collapse to trivial solutions. The penalty weight *λ* serves as a regularization parameter here that controls the strength of group assignment balancing without being tied to specific constraints of the model architecture or dataset characteristics, similar to weight decay or learning rate parameters in standard optimization routines. The value for *λ* can therefore be chosen through standard hyperparameter optimization techniques such as grid search, random search, or Bayesian optimization methods.

Here, we also found that using appropriately slow learning rates also promotes stability, as excessively large learning rates can effectively “overpower” the penalty function. Aside from this, we have not observed notable changes in computational cost or efficiency compared to other common loss functions, such as mean squared error or binary cross-entropy.

To validate our proposed framework, we implemented varying experimental approaches to test our methodology across diverse data modalities and neural network architectures.

First, we conducted a simulation experiment with synthetic tabular data where we established ground truth groupings and systematically evaluated a MLP’s ability to recover these predefined patient clusters. Second, we applied a CNN in analogous simulation experiments to cluster handwritten digits based on associated simulated survival times, establishing ground truth recovery performance in an optical recognition setting. In both cases, the MLP and CNN achieved area under the receiver operating characteristic curve (AUROC) values of 0.94 ± 0.01 and above and area under the precision-recall curve (AUPRC) values of 0.91 ± 0.01 and above at recovering predefined patient groupings in an unsupervised manner through survival-guidance alone (detailed in Table 1; refer to Supplementary Results section *Performance assessment of our method on simulated data* for details).

**Table 1:** Performance metrics (AUROC and AUPRC) in simulations using a MLP to cluster synthetic feature vectors and a CNN to cluster handwritten digits based on associated survival times, respectively

	MLP	CNN
**AUROC**		
class 0	0.96 ± 0.00	0.97 ± 0.00
class 1	0.96 ± 0.01	0.94 ± 0.01
class 2	0.96 ± 0.00	0.98 ± 0.00
**AUPRC**		
class 0	0.92 ± 0.01	0.94 ± 0.01
class 1	0.92 ± 0.01	0.91 ± 0.01
class 2	0.93 ± 0.01	0.95 ± 0.01

Values show the mean ± s.d. obtained through 5-fold cross-validation.

Building on these simulation studies, we next sought to evaluate the generalizability of our approach by applying it to real-world clinical datasets from two distinct tumor entities (one hematologic and one solid malignancy), each characterized by entirely different input modalities: laboratory parameters in the former and CT scans in the latter. To this end, we employed a MLP to cluster MM patients based on laboratory blood parameters, evaluating its performance in separating patients into prognostically distinct risk groups based on overall survival. We conducted explainability analyses to identify which laboratory parameters most strongly influenced the model’s clustering decisions to gain insight into the prognostic features leveraged for patient stratification. Finally, we advanced to cluster non-small cell lung cancer (NSCLC) patients based on CT imaging data using a CNN, achieving significant separation in survival outcomes. We further employed visualization techniques to identify attention patterns within the images that drove the model’s decisions, revealing morphological features associated with prognosis that align with conventional staging approaches.

The results of our analyses are detailed below.

### Unsupervised risk stratification of MM patients using routine blood work

**Fig. 1:**
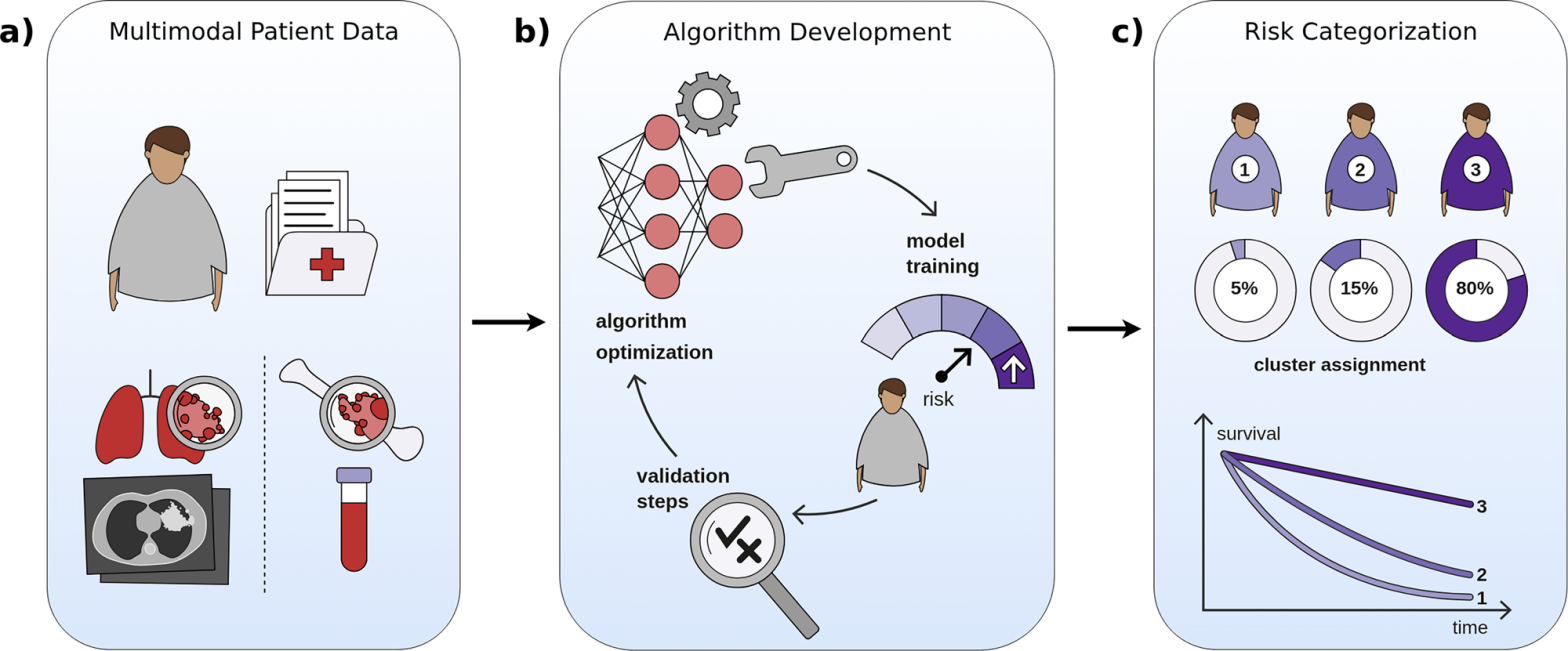
Graphical abstract of the machine learning pipeline for patient survival clustering. The workflow progresses from raw patient data collection through algorithmic processing to final risk classification to enable personalized prognostic assessment based on multivariate clinical parameters. **a** Input data comprising multimodal clinical patient data such as laboratory parameters and genetic information. **b** Development phase showing the implementation of a custom machine learning algorithm. The panel includes algorithm optimization (top), model training (lower right), and validation steps (lower left). **c** Visualization of the desired model output displaying survival outcomes of patient risk clusters and partial attribution of patients to each group.

**Fig. 2:**
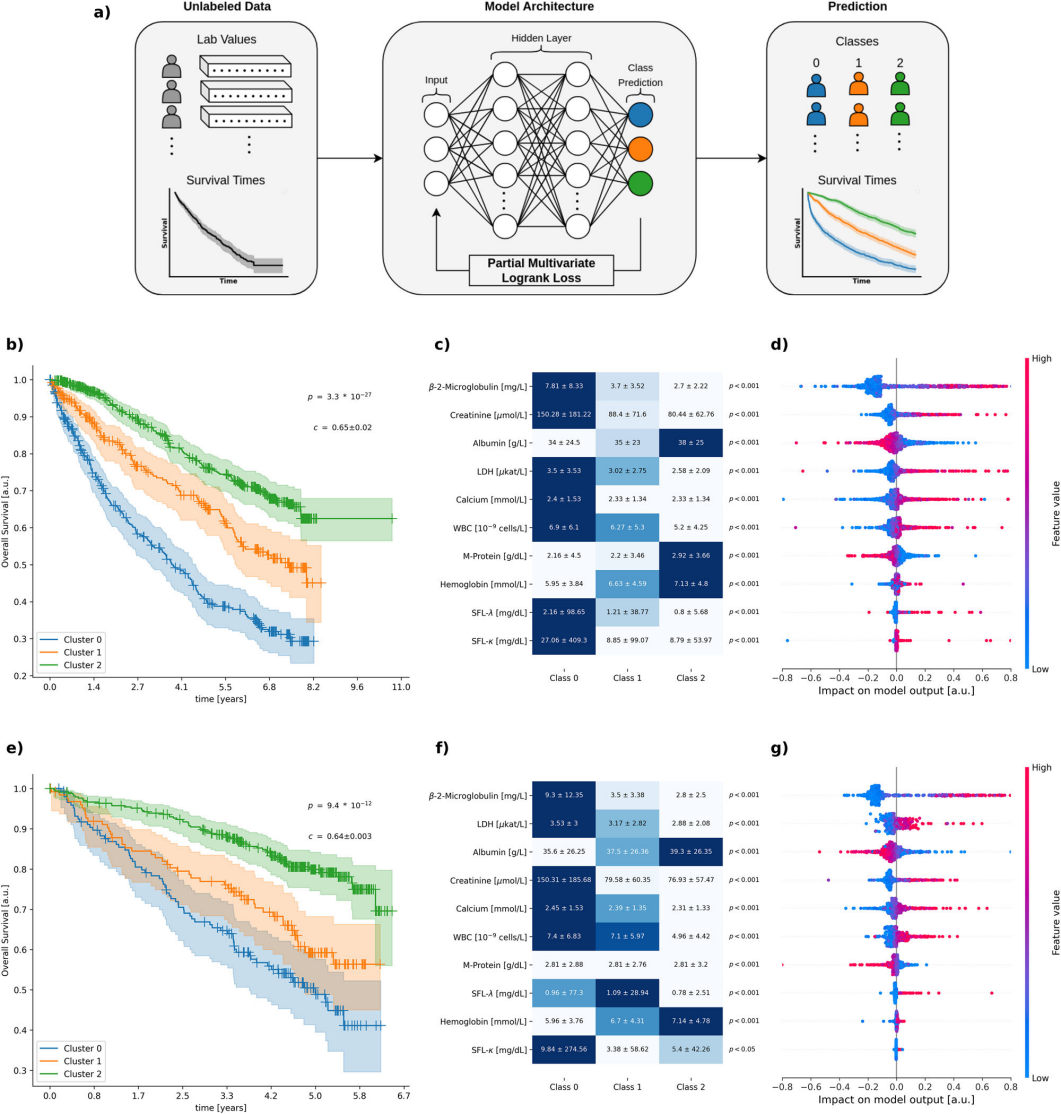
Application of our method by stratifying MM patients based on biomarker profiles. **a** Workflow demonstrating unlabeled patient data (left) encompassing lab values and survival times processed by a MLP (center) trained on our custom *PartialMultivariateLogrankLoss* to generate class predictions (right) categorized into three classes (0, 1, 2) with corresponding survival time curves. **b** Overall survival analysis showing significant stratification of CoMMpass patients into three distinct risk clusters (*p* = 3.3 × 10^−27^, *c* = 0.65 ± 0.02). Cluster 0 (blue) demonstrates the poorest outcomes with median survival at approximately at 4 years, Cluster 1 (orange) shows intermediate outcomes, while Cluster 2 (green) shows the most favorable prognosis with approximately 70% survival rate at 9 years. **c** Biomarker distribution (mean ± s.d.) across the three patient classes for laboratory parameters *β*-2-Microglobulin, Creatinine, M-Protein, Calcium, Albumin, LDH, WBC, Hemoglobin, SFL-*κ*, and SFL-*λ*. Highly significant differences (*p* < 0.001) were observed for all parameters based on a Kruskal-Wallis test across the three classes. Color gradients (light to dark) visually represent feature’s mean values within each group (low to high respectively) normalized by each parameter’s range. **d** Feature importance analysis showing the impact of biomarkers on model output, with beeswarm plots displaying SHAP values for the biomarkers shown in panel c at the same height. The visualization uses a color gradient (blue to red) to indicate feature values from low to high, with dots showing the distribution of impact values across the patient population. All shown data in panels **b,** **c** & **d** were derived from the testing partitions of the 5 cross-validation-folds and are entirely based on unseen patients. **e** External validation on GMMG-MM5 dataset showing overall survival analysis with significant stratification of patients into three distinct risk clusters (*p* = 9.4 × 10^−12^ *c* = 0.64 ± 0.003). **f** Biomarker distribution (mean ± s.d.) across the three patient classes in the GMMG-MM5 dataset for the same laboratory parameters as shown in panel c. Significant differences were observed for all parameters based on a Kruskal-Wallis test across the three classes. Color gradients (light to dark) visually represent feature’s mean values within each group (low to high respectively) normalized by each parameter’s range. **g** Feature importance analysis on GMMG-MM5 dataset showing the impact of biomarkers on model output, with beeswarm plots displaying SHAP values for the biomarkers shown in panel **f** at the same height. All shown data in (**e**)–(**g**) demonstrate external validation of the model on the external GMMG-MM5 dataset, confirming the generalizability of the identified risk stratification patterns to independent contexts.

To demonstrate the clinical utility of our optimization framework, we next applied it to stratify MM patients based solely on routine blood work parameters (Figs. 1 and 2a). We utilized the CoMMpass dataset, one of the largest publicly available clinical MM resources, containing comprehensive biomarker profiles alongside long-term outcomes, enabling the evaluation of our risk stratification approach.

Furthermore, MM was selected as the real-world use case due to the availability of well-established risk stratification systems developed over the past decades, specifically the R-ISS^10^. The inclusion of MM thus enables us to benchmark our method’s ability to recover established, biologically relevant prognostic features such as *β*-2-microglobulin (*β*2m), Creatinine (Cr), Albumin (Alb), and others.

Based on these considerations, we selected ten biomarkers routinely collected in clinical practice and known to reflect the disease biology of MM (refer to methods section *Patient characteristics and data preprocessing* for details and rationale). A MLP model was trained to cluster patients into three distinct risk groups based on these parameters (Fig. 2a).

In accordance to best practices in machine learning, we partitioned the dataset using 5-fold cross-validation and would exclusively evaluate model performance in unseen patients belonging to the withheld testing partitions (Table 3). The model achieved a highly significant separation of the overall survival (*p* < 10^−26^, *c* = 0.65 ± 0.02, Fig. 2b), with distinct survival trajectories across the three clusters. Cluster 0 exhibited the poorest prognosis with median survival of approximately 4 years, Cluster 1 demonstrated intermediate outcomes, while Cluster 2 showed markedly better prognosis with approximately 70% survival at 9 years.

Notably, our model achieved a c-index of *c* = 0.65 ± 0.02, higher than the c-index for the R-ISS in MM, which amounted to 0.62 ± 0.03 in the CoMMpass cohort, aligning well with the typically reported values of 0.61^25,26^ for different cohorts.

This performance is particularly significant considering that the R-ISS relies on cytogenetic data, which currently are among the strongest known prognostic factors in MM, whereas our approach utilizes only blood parameters that accumulate naturally during standard clinical care.

Analysis of biomarker distributions across the three identified clusters revealed clinically meaningful patterns that align with established disease biology (Fig. 2c, refer to Supplementary Results section *Supplementary results of the MM application* for details). All biomarkers showed statistically significant differences across the three clusters based on a Kruskal-Wallis test.

Feature importance analysis using shapley additive explanation (SHAP) values (Fig. 2d) provided further insights into the model’s decision-making process. Here, *β*2m and Cr emerged as the most influential parameters, with higher SHAP values (red) being associated with higher risk, consistent with their respective roles in renal dysfunction and disease progression in MM. Elevated Alb and Hemoglobin (Hb) showed association with lower risk and improved outcomes, which may reflect the adverse impact of systemic inflammation or impaired protein synthesis and anemia on patient prognosis. These patterns align well with clinical expectations and established disease biology, providing compelling validation of our method to uncover biologically relevant risk patterns.

Notably, our approach identified these patient subgroups without any prior knowledge of established risk factors or staging systems. The model learned to recognize the patterns in the biomarker data solely through optimization of survival heterogeneity between the identified clusters.

To evaluate the performance of our clustering approach beyond the three-cluster configuration, we performed an additional layer of analyses varying the number of clusters *k* (Supplementary Figure 7a& b). Specifically, we trained models to cluster patients into *k* = 4 and *k* = 5 groups. Both the four-cluster (*k* = 4, *p* < 10^−22^, *c* = 0.65 ± 0.04) and five-cluster (*k* = 5, *p* < 10^−25^, *c* = 0.66 ± 0.05) configurations maintained highly significant risk stratification with c-indices of comparable magnitude to our primary three-cluster model. While the additional clusters revealed potential for further granularity in risk assessment, with the highest-risk groups showing the most pronounced survival declines, we observed that for *k* = 5, clusters began to overlap with one another, as evidenced by the converging survival curves of clusters 2 and 3 (Supplementary Fig. 7b). This indicated to us that beyond an optimal cluster number, additional partitions tend to subdivide existing risk groups without providing meaningful prognostic improvement.

To assess the generalizability of our clustering approach, we performed external validation on the independent GMMG-MM5 study dataset^27^, which comprises a distinct patient cohort (Fig. 2e–g).

Here, the constructed model maintained robust prognostic performance in this external context (*c* = 0.64 ± 0.003), achieving significant risk stratification (*p* < 10^−11^, Fig. 2e). Importantly, the biomarker distribution patterns across clusters as well as the feature importance rankings based on SHAP analysis remained consistent with the internal validation cohort (Fig. 2f, refer to Supplementary Results section *Supplementary results of the MM application* for details). Here, the successful external validation in a different patient population reinforces confidence in the transferability of our clustering methodology across clinical settings and its potential for broader clinical application.

To contextualize the performance of our proposed framework, we compared our method against several established baseline techniques on both our internal and external validation cohorts (Table 2). To this end, we selected the Random Survival Forest^3^ and the Penalized Cox model^28^ as a baseline for comparison with survival-guided methods. Additionally, we included the recently proposed Neural Survival Clustering (NSC)^16^ model as a baseline for nonlinear representation learning combined with survival information. Before evaluating it’s performance, we tuned the hyperparameters of the NSC model via Bayesian optimization using optuna^29^, analogously to the MLP (refer to methods section *Model implementation and training* for details). We also included K-Means and Gaussian Mixture models as unsupervised clustering alternatives, without any survival-guided optimization. The R-ISS was additionally included in the comparative analysis as the current clinical standard for MM prognosis^30^.

**Table 2:** C-indices for internal validation on CoMMpass dataset and external validation on GMMG-MM5 dataset using different baseline comparators

	Internal validation	External validation
	on CoMMpass dataset	on GMMG-MM5 dataset
MLP constructed with our method	0.65 (0.63 — 0.68)	0.64 (0.60 — 0.68)
Random Survival Forest	0.65 (0.62 — 0.67) n.s.	0.64 (0.60 — 0.68) n.s.
NSC	0.64 (0.62 — 0.67)^†^	0.61 (0.57 — 0.64)*
Penalized Cox-Model	0.63 (0.61 — 0.66)*	0.65 (0.61 — 0.69) n.s.
R-ISS	0.62 (0.59 — 0.65)*	0.60 (0.54 — 0.66)*
Gaussian Mixture	0.58 (0.56 — 0.61)*	0.56 (0.52 — 0.59)*
K-Means	0.56 (0.54 — 0.59)*	0.58 (0.55 — 0.62)*

Values are reported as median and 95% confidence intervals obtained from nonparametric bootstrap resampling of the cohorts using cross-validated predictions of each estimator. Symbols ^†^ and * denote *p* < 0.1 and *p* < 0.05, respectively, inferred from a bootstrap distribution of the paired difference in performances between the MLP and each respective estimator (refer to methods section Statistics for details).

To compare estimators, we conducted hypothesis testing via pairwise comparisons between the MLP and each comparator based on the nonparametric bootstrap distribution of paired differences in model performance (refer to methods sectionModel implementation and training for details). Notably, the MLP constructed using our proposed method performed en par with the Random Survival Forest in the internal validation on CoMMpass and en par with both the Random Survival Forest and Penalized Cox-Model in the external validation GMMG-MM5. The MLP outperformed all other baseline estimators with statistical significance (*p* < 0.1 for NSC in internal validation, *p* < 0.05 in all other instances, Table 2), which included the R-ISS in both internal and external validation.

Lastly, to test the limits of our methodology, we sought to apply it to data characterized by very high dimensionality. Specifically, we used bulk mRNA sequencing data from bone marrow plasma cells comprising over 226,654 transcripts (supplementary results section *Transcriptomics model implementation and training*). Here, the model obtained using our method successfully stratified patients into six distinct prognostic clusters with decent discrimination (*c* = 0.67 ± 0.04) and highly significant separation (*p* < 10^−16^; Supplementary Figure 6a). Top ten transcripts driving assignment to both high-risk (Cluster 0) and low-risk (Cluster 5) groups, were investigated using SHAP. These determinants included transcripts that align with clinical expectations (e.g. immunoglobulin lambda variable 3-29 (IGLV3-29-201), Immunoglobulin kappa variable 1D-17 (IGKV1D-17-201), Cancer susceptibility 15 (CASC15-254), Fibroblast growth factor receptor 2 (FGFR2-210), etc.) as well as several novel, not yet characterized transcripts (Supplementary Fig. 6b and c).

### Uncovering prognostic signatures in CT images of NSCLC patients

Building on the encouraging results in MM, we next sought to demonstrate the versatility of our optimization framework by applying it to complex medical imaging data, where techniques for survival-guided clustering and extraction of features driving differential prognosis remain sparse (refer to Discussion for detailed survey).

To this end, we developed a complementary approach for stratifying NSCLC patients based solely on CT imaging data, maintaining our core methodology while changing the network architecture to a CNN to suit this different data modality.

We applied our method to the Lung1 dataset^31^, comprising pretreatment CT scans from 422 NSCLC patients with documented clinical outcomes. This dataset is particularly suitable for validating our approach as it contains a diverse range of tumor phenotypes and has been extensively studied for prognostic radiomics signatures^32^, thus providing a robust benchmark for our methodology.

**Fig. 3:**
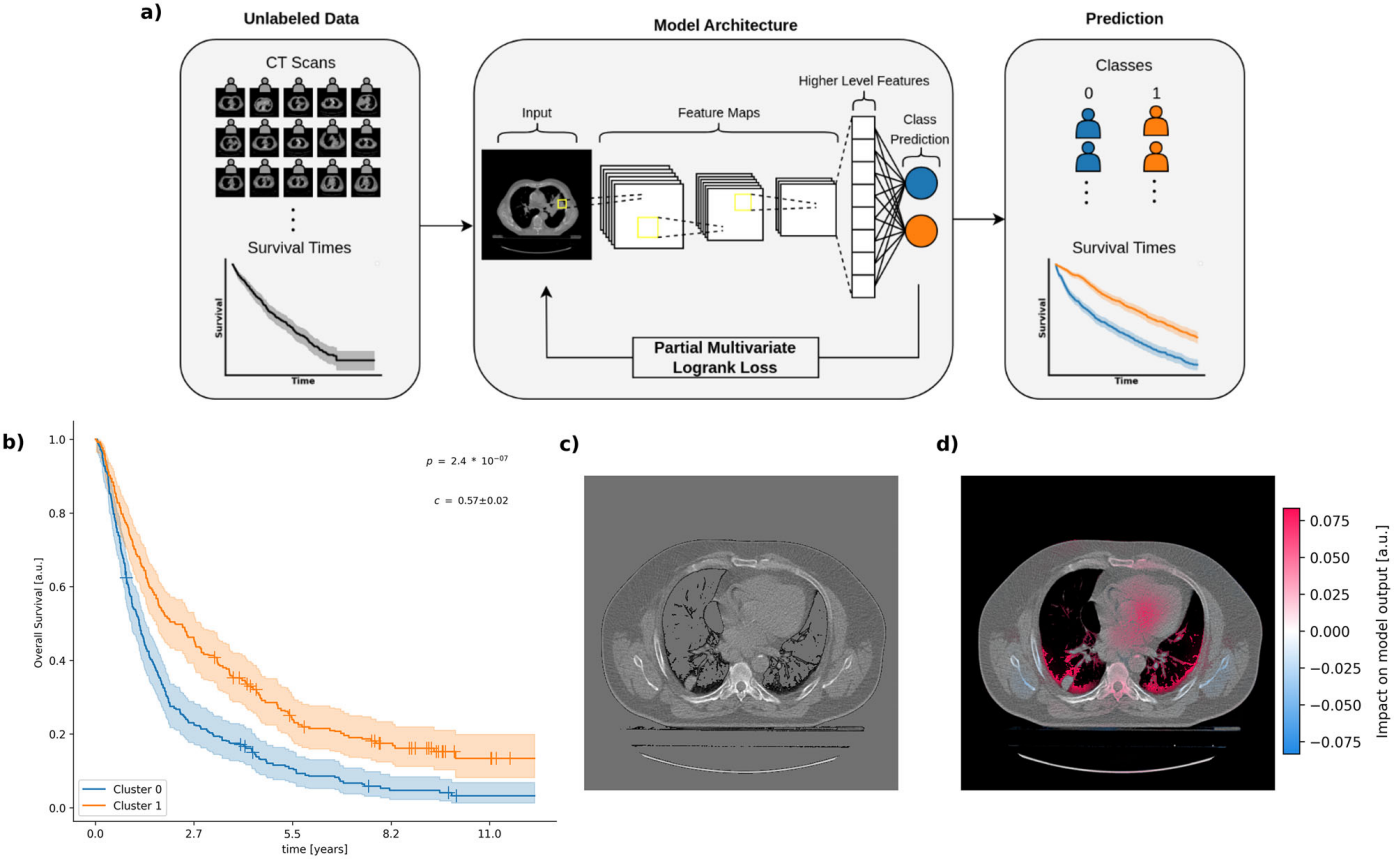
Application of our method by stratifying NSCLC patients based on CT imaging data. **a** Radiomics workflow demonstrating the application of a CNN to CT images for classifying NSCLC patients into distinct risk groups (high vs. low) based on our custom PartialMultivariateLogrankLoss. **b** Kaplan-Meier survival curves showing significant difference (*c* = 0.57 ± 0.02, *p* = 2.4 × 10^−7^) between the two patient clusters identified by the radiomics model. Cluster 0 (blue) exhibits poorer survival outcomes compared to Cluster 1 (orange). **c** Representative CT image with zero-signal preserving contrast enhancement. **d** SHAP value heatmap corresponding to CT image in panel c, illustrating pixel-level importance for the model’s survival-based predictions. Color scale bars indicate SHAP value magnitude (arbitrary units), representing increased model attention in colored areas. Red areas indicate features positively associated with high-risk classification, while blue areas represent features associated with lower risk. All shown data were derived from the testing partitions of the 5 cross-validation-folds and are entirely based on unseen patients.

We trained a custom CNN on 2D axial slices of the thoracic CT scans to cluster patients into two risk groups (Fig. 3a). Critically, our approach required no manual tumor segmentation or preprocessing beyond basic image normalization (see methods section *Patient characteristics and data preprocessing*). The CNN was trained using the same *PartialMultivariateLogrankLoss* function employed in our previous experiments, optimizing for maximum between-group survival heterogeneity without any prior knowledge of malignancy, tumor identity, or location. This “raw-data” approach is rather distinct from to traditional radiomics methods that typically require extensive manual preprocessing and feature engineering.

Like before, we partitioned the dataset using 5-fold cross-validation and would exclusively evaluate model performance in unseen patients belonging to the withheld testing partitions (Table 4). The resulting patient stratification achieved a highly significant separation of survival curves (*c* = 0.57 ± 0.02, *p* < 10^−6^, Fig. 3b), with Cluster 0 showing markedly poorer outcomes compared to Cluster 1. The median survival times for cluster 0 and cluster 1 were 446 days and 806 days, respectively, demonstrating performance comparable to the performance reported in the original radiomics analysis by Aerts et al.^32^, where median survival times were approximately 400 days and 750 days respectively, despite our method requiring substantially less manual preprocessing and feature engineering. Analogous to our evaluations in MM, we invesstigated the performance of our clustering approach beyond the two-cluster configuration, by training CNNs to cluster patients into *k* = 3 and *k* = 4 groups (Supplementary Fig. 7c, d). Again, both configurations maintained significant risk stratifications (*k* = 3, *p* < 0.01, *c* = 0.54 ± 0.01; *k* = 4, *p* < 0.001, *c* = 0.55 ± 0.05). As before, we would observe that for *k* = 4, clusters began to overlap, as evidenced by the convergence of survival curves of clusters 0 and 1 and cluster 2 and 3 (Supplementary Fig. 7d), reinforcing that additional partitions tend to subdivide existing risk groups without providing additional prognostic improvement.

To understand how the model made its decisions, we performed post-hoc SHAP explainability analyses (Fig. 3c, d). Remarkably, despite being trained without any tumor annotations or location information, the model’s attention was predominantly focused on the intrapulmonary region of the thorax, specifically on areas containing tumor infiltrates. This observation provides evidence for our method’s ability to train models to autonomously identify clinically relevant patient features even in data modalities as complex as CT imaging data.

**Fig. 4:**
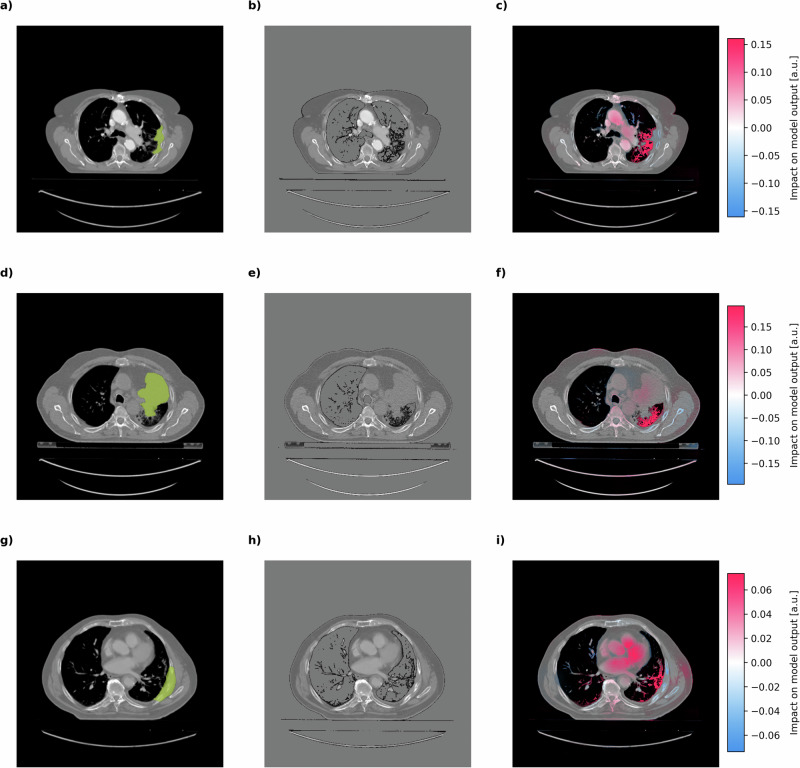
Co-localization of human-annotated tumor regions with CNN attention patterns in high-risk NSCLC patients. CT scan slices from three representative NSCLC patients (**a**–**c**, **d**–**f**, and **g**–**i**) showing: manual tumor annotations by clinical experts highlighted in green (left column: **a**, **d**, **g**); reference CT images with zero-signal preserving contrast enhancement (middle column: **b**, **e**, **h**); and corresponding SHAP value heatmaps (right column: **c**, **f**, **i**) from the CNN trained to cluster images based on patient survival time. The CNN was trained without access to human tumor annotations. SHAP values represent pixel-level importance for the model’s survival-based predictions. Color scale bars indicate SHAP value magnitude (arbitrary units), representing increased model attention in colored areas. Note the remarkable co-localization between expert-identified tumor regions and areas of peak CNN attention, demonstrating that the survival prediction model independently learned to focus on clinically relevant tumor features. All shown data were derived from the testing partitions of the 5 cross-validation-folds and are entirely based on unseen patients.

We further validated these findings by comparing the CNN’s attention patterns with human expert annotations of tumor masses (Fig. 4). These annotations are available in the Lung1 dataset but were deliberately withheld from the model during training. The comparison revealed robust co-localization between expert-identified tumor regions and areas of peak CNN attention, specifically growth patterns in the immediate tumor environments. This notable alignment implies that our approach successfully learned to identify tumor morphology features without labeling through survival-guidance alone.

Intriguingly, detailed analysis of the attention patterns revealed that the model did not simply recognize the tumor mass directly, but rather focused on the branching infiltrative tissue originating from the tumors (Fig. 4c, f, i). This finding aligns with the clinical understanding of NSCLC progression, where infiltrative growth patterns and adjacent interstitial lung abnormalities (ILA)^33^ are associated with more aggressive disease^34–36^. In high-risk patients, these infiltrative regions display predominantly positive (red) SHAP values, indicating their association with poorer outcomes.

Interestingly, in some instances, the model’s attention patterns also included cardiac vessels for assignment to the high-risk cluster, as evidenced by the patient in Fig. 4i. The model’s attention to cardiovascular features here may reflect that the model learned to integrate cardiac imaging biomarkers for high-risk classification, which are known to have prognostic value^37,38^. This finding is consistent with the established association between NSCLC and cardiovascular disease and the increased risk of cardiovascular events, diminishing overall survival in affected patients^39–41^.

**Fig. 5:**
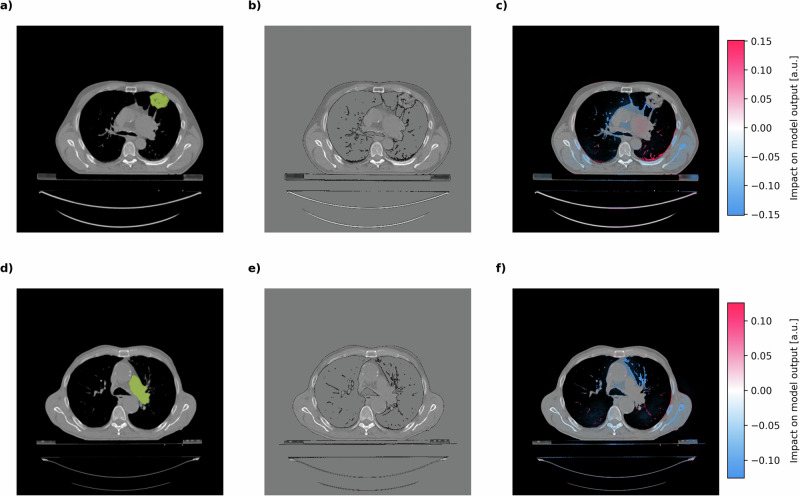
Co-localization of human-annotated tumor regions with CNN attention patterns in low-risk NSCLC patients. CT scan slices from two representative low-risk NSCLC patients (**a**–**c** and **d**–**f**) showing: manual tumor annotations by clinical experts highlighted in green (left column: **a**, **d**); reference CT images with zero-signal preserving contrast enhancement (middle column: **b**, **e**); and corresponding SHAP value heatmaps (right column: **c**, **f**) from the CNN trained to cluster images based on patient survival time. The CNN was trained without access to human tumor annotations. SHAP values represent pixel-level importance for the model’s survival-based predictions. Color scale bars indicate SHAP value magnitude (arbitrary units), representing increased model attention in colored areas. In contrast to high-risk patients, these low-risk cases demonstrate different attention patterns from the CNN, with predominantly negative (blue) SHAP values in tumor regions. This suggests the model identifies distinct radiographic features in tumors associated with better prognosis, highlighting the CNN's ability to differentiate survival-relevant characteristics beyond simple tumor detection. All shown data were derived from the testing partitions of the 5 cross-validation-folds and are entirely based on unseen patients.

Conversely, in patients classified as low-risk (Fig. 5), the model maintains its focus on tumor-adjacent tissue with the lesser branching in tumor morphology resulting in predominantly negative (blue) SHAP values. This suggests the model identifies radiographic features associated with better prognosis, such as more contained growth patterns and less extensive infiltration. The contrast between attention patterns in high-risk versus low-risk patients demonstrates the model’s ability to differentiate survival-relevant characteristics beyond simple tumor detection.

To further validate our CNN-based approach, we applied the trained model to CT scans from NSCLC patients treated at our institution, representing an independent clinical context with different imaging protocols and patient demographics (Supplementary Fig. 8). Consistent with our previous observations, the model demonstrated attention patterns that align with established clinical knowledge. Here, we could reproduce the CNN’s focus on infiltrative growth patterns emerging from tumor tissue (Fig. 8a–c), where irregular, spiculated margins and heterogeneous density patterns contributed positive (red) SHAP values associated with poor prognosis^34–36^. Furthermore, in some instances the model again incorporated cardiac regions into its risk assessment (Supplementary Fig. 8d–f), reinforcing the clinical relevance of cardiovascular comorbidity in NSCLC prognosis as previously discussed^39–41^. Notably, the model attributed negative SHAP values (blue regions) to the intra-pulmonary regions for patients with well-contained, circumscribed tumors (Supplementary Fig. 8g–i), suggesting that more localized growth patterns without infiltrative characteristics were associated with favorable outcomes. These consistent attention patterns across independent patient cohorts demonstrate the robustness of our approach and its ability to identify clinically meaningful imaging features that generalize across institutions.

These findings are noteworthy considering the solely survival-guided nature of our approach. Without any prior knowledge of tumor biology or radiographic features, the model autonomously learned to recognize higher degrees of branching infiltrative tumor growth and cardiac morphology as risk factor in NSCLC patients, which are known adverse prognostic markers^34,38^.

Here, the external validation reinforces confidence in the robustness of models constructed using our approach, especially since radiomics features are known to be notoriously hard to generalize across different clinical sites^42,43^.

## Discussion

In this study, we introduced a novel optimization framework for training arbitrary neural networks to identify prognostically distinct patient subgroups across diverse cancer types and data modalities. By formalizing the core optimization problem of survival-based clustering without prior knowledge of class labels, we have proposed a universal framework that enables (1) optimal stratification of patients into prognostically distinct subgroups (2) flexibly across diverse clinical input modalities and (3) irrespective of model architecture. Our validation across distinct scenarios (application to both tabular- and image-based synthetic data, real-world biomarker data of MM patients and CT imaging data of NSCLC patients), as well as external validation in independent cohorts for the real-world applications, demonstrates the versatility and effectiveness of our approach. Moreover, we demonstrated that, in the real-world applications, our method successfully identified prognostically relevant patterns with minimal to no preprocessing and without prior knowledge of established risk factors.

A central challenge in developing unsupervised approaches of any kind but especially for patient stratification is the absence of definitive ground truth to validate obtained clustering against^44^. In clinical applications, we can assess statistical separation of resulting survival distributions or if these are based on clinically interpretable features, as we did in our applications to MM and NSCLC data, but cannot definitively determine whether the identified groups represent the optimal stratification. Our simulation experiments with synthetic data provided a crucial validation step for our framework by creating a ground truth association between specific input features and survival outcomes, allowing us to systematically evaluate the frameworks’ ability to enable models to recover these predefined relationships. The performance of our approach in these controlled settings (achieving AUROC and AUPRC values above 0.90 for both tabular and image data) provided the necessary evidence for our optimization framework being able to readily identify features that are associated with prognostically distinct patient subgroups.

Her, our model’s performance in MM risk stratification is encouraging, as it recapitulates known clinical associations while achieving similar performance to established staging systems using only routine blood parameters.

The model’s ability to surpass the performance of the R-ISS (c_MLP_ = 0.65 (CI: 0.63 — 0.68) vs. c_R-ISS_ = 0.62 (CI: 0.59 — 0.65) in CoMMpass, c_MLP_ = 0.64 (CI: 0.60 — 0.68) vs. c_R-ISS_ = 0.60 (CI: 0.54 — 0.66) in GMMG-MM5 and c = 0.61 in literature^25,26^) with statistical significance is notable, given that the R-ISS incorporates cytogenetic abnormalities^10^, which are among the strongest known prognostic factors in MM.

This finding suggests that machine learning approaches can extract comparable or even superior prognostic information from routine blood parameters alone, potentially reducing the need for specialized testing in some clinical scenarios and enabling risk stratification in resource-limited settings where cytogenetic testing may be unavailable or non-economical. Moreover, while the role of well-known risk factors in established clinical staging systems including *β*2m, Alb, Cr, and Lactate dehydrogenase (LDH)^45^ were reproduced, the model also leveraged biomarkers that extend beyond established clinical staging. Here, the distribution of serum free light-chain (SFL)s across the clusters demonstrates the potential of our approach, with the high-risk cluster showing significantly elevated levels compared to the favorable-risk cluster (Fig. 2c), which might reflect the known association between elevated free light chains and aggressive disease biology^46,47^, including higher rates of renal impairment and faster disease progression^46,48^.

The external validation of our framework on the independent GMMG-MM5 study dataset^27^, which comprises a distinct patient cohort (Fig. 2e-g) further confirmed the generalizability of our approach. Here, the constructed models maintained robust prognostic performance in the external context, achieving significant risk stratification with biomarker distribution patterns across clusters remaining consistent with the internal validation result and aligning with established clinical knowledge. The successful external validation in a different patient population reinforces confidence in the transferability of our clustering methodology across clinical settings and its potential for broader clinical implementation.

Having established the validity of our clustering methodology in the context of our MM-specific application, our blood-based model naturally invites comparison with recently developed prognostic systems in MM. A notable example of such contemporary benchmarks is IRMMa^49^. IRMMa is a prognostic system that, within a multi-state model framework, integrates genomic features alongside treatment variables, and, perhaps most importantly, longitudinal follow-up data that captures the evolution and progression of the disease, which is among the most important determinants of survival in MM^50^. Here, it is worthwhile to distinguish between our methodological objective and the development of comprehensive clinical prognostic tools. Importantly, our primary objective with the present work was not to propose a competing clinical prognostic tool, but a construction method for deep-learning models adept at identifying risk factors before treatment initiation and to validate it by applying it to different data modalities of different cancer entities with known prognostic relevance. Here, one such application-case was the most recent snapshot of multiple myeloma lab data after diagnosis.

Consequently, IRMMa would undoubtedly demonstrate superior performance in a comparative setting, which would reflect its greater model-complexity and richer data integration rather than the methodological contribution we set out to evaluate here. A fair comparison would hence require constructing a more dedicated competing model, which leverages an equally great spectrum of genomic- and treatment variables and longitudinal follow-up data. We acknowledge IRMMa’s greater sophistication and its superior prognostic utility when genomic and longitudinal data are available; Integrating the here proposed method with longitudinal and genomic inputs toward a multi-state framework in the spirit of IRMMa is indeed a promising prospect for future work.

Consequently, a promising future direction in MM would be the application of our method to genomic or single-cell sequencing data, specifically in the recently emerging therapeutic area of CAR T-cell therapy where risk stratification approaches continue to be refined^51,52^ but are still in their infancy.

While in MM our proposed optimization framework has been applied to tabular data, it is important to note that our methodology can be applied to data from various modalities beyond tabular data, as we have demonstrated through our application to CT imaging data in NSCLC patients.

While our method performs en par with Random Survival Forests and Penalized Cox-Model in tabular data, our proposed method offers the advantage of plug-and-play compatibility with arbitrary neural architectures and thus of having a single methodological framework that can be applied to tabular clinical data, clinical imaging, and potentially other modalities as well (e.g. time series data using LSTMs, or unstructured clinical reports using Transformer models). Our application to CT imaging data in NSCLC patients underscores the versatility of our framework in this regard. Notably, the baseline methods we included for comparison are specifically designed for tabular data and cannot be readily applied to imaging data or other data types. Indeed, bringing in a suitable comparator for our imaging-based application remained challenging, as techniques for survival-guided clustering and extraction of features driving differential prognosis in the realm of medical imaging are generally sparse.

To clarify, while related ideas exist in literature, imaging-based survival-clustering methods can generally be grouped into two main approaches: Firstly, images are clustered (without survival guidance) and the resulting clusters are tested for coinciding with outcomes (which they may or may not). Examples include De Simone et al.^53^, Espis et al.^54^, Lee et al.^55^, and Mackenzie et al.^56^. However, as the prior clustering is blind to survival outcomes, there is no guaranteed prognostic relevance of the identified patient subgroups, even though these groups may be distinct in other regards. Secondly, image features are extracted, either via pretrained/self-supervised deep-learning models or manual radiomics, and the resulting tabular features are subjected to traditional survival analysis post-hoc (examples include Ghesu et al.^57^, Guy et al.^58^, Schön et al.^59^, Wessels et al.^22^, Tang et al.^21^). While feature-extraction combined with post-hoc survival-analysis proves powerful in most scenarios, the primary feature extraction is again blind to outcomes and may thus miss survival-specific signals or yield signals irrelevant to outcomes. Furthermore, as the extracted features are subjected to traditional survival-analysis, this approach cannot be considered as clustering in a strict sense, as it lacks providing decision boundaries that facilitate straightforward stratification into distinct risk groups^4^. A recent review^60^ also notes that end-to-end survival modeling directly from images remains challenging.

Beyond the cited literature, and given their constraints, we are not aware of any approaches that offer clustering of imaging data in a directly survival-guided manner at all. Here, we believe our approach to address this knowledge gap by providing a method that directly optimizes for image-derived features that align with prognostically different patient groups.

Without any tumor annotations or location information during training, our method was able to autonomously learn to focus on clinically relevant regions, with attention patterns that showed notable co-localization with expert-identified tumor areas, while focusing on prognostically relevant tumor morphology. This ability to autonomously recognize infiltrative tumor tissue growths and ILA, which are well-known risk factors in NSCLC^33,36,61–63^, represents a level of feature learning that goes beyond simple tumor detection. This finding demonstrates that survival outcomes alone can guide neural networks to discover clinically meaningful imaging biomarkers without explicit supervision. To extract such clinically interpretable imaging features in a solely survival-guided manner represents a significant advancement in the application of artificial intelligence in cancer^64–66^. Here, our approach both achieves robust prognostic performance and provides explainable results that could complement clinical decision-making and potentially reveal novel imaging biomarkers.

The external evaluation using CT imaging data from NSCLC patients treated at our institution reinforces confidence in our approach in the context of image analysis. The attention patterns in the external cohort showed similar focus on tumor- and cardiac morphology, and ILA, reinforcing the generalizability of the identified imaging biomarkers.

These results are encouraging given the known challenges in radiomics feature reproducibility across different imaging protocols and institutions^42,43^. The ability to reproduce these observations across different datasets suggests that the learned representations indeed capture biological factors associated with NSCLC disease burden and are robust against scanner- or population-specific biases.

Despite the promising findings, several limitations of our study warrant discussion. It is important to emphasize that, in the research presented here, we were not aiming at proposing a competing disease-specific clinical model but to develop and validate a model-creation framework for broad applicability of artificial intelligence in cancer care. Notably, we could identify well-known clinical parameters reflecting disease burden in MM and radiographic features in NSCLC that are well-known to be associated with poor outcomes, which provides evidence of our methodology’s utility. Importantly, as the prognostic relevance of these features is already well-characterized, the primary contribution of our work lies not in the identification of these features itself, but in the development of an approach that enables autonomous feature discovery in cancer research, which we validated by reproducing these established risk factors. Moreover, our approach currently relies on post-hoc interpretability methods like SHAP analysis to understand model decisions. Future work that focuses on a specific modeling task could explore integrating interpretability directly into the architecture of the respective models to avoid the need for post-hoc analyses and provide more readily available explanations of the model outputs. Third, in our current implementation, the optimal number of risk groups must be specified a priori. Determining the optimal number of clusters is a common challenge in unsupervised clustering problems. While we drew on clinical knowledge to inform our choices for cluster numbers in the presented use cases, determining the optimal number of clusters in a setting where clinical knowledge is limited should be guided by elbow methods^67^, a dirichlet process^68^, or other similar techniques. Finally, while in the applications we presented here, each data modality was treated separately, integrating multiple data types (e.g., imaging, genomics, and clinical parameters) into a unified multi-modal model could potentially enhance prognostic accuracy and provide more comprehensive patient characterization, which is a promising direction for future work.

Furthermore, it is important to note that while our approach does not require pre-labeled patient categories or explicit supervision regarding which patients belong to which risk groups, it utilizes survival outcomes to guide the discovery of latent patient groupings. Moreover, our method leverages survival information as an optimization signal to uncover hidden structure within the data, allowing the model to autonomously identify patient subgroups based on patterns that maximize survival heterogeneity. This form of optimization must therefore be considered a hybrid approach that combines the exploratory nature of unsupervised learning with the clinical relevance of survival-guided optimization.

Traditional cancer classification systems like R-ISS and TNM Classification of Malignant Tumors (TNM) staging, while foundational to clinical practice, struggle to adapt to the complexities of modern oncology^69^. These systems typically rely on a limited number of predefined risk factors, which may not capture the full spectrum of disease heterogeneity or the complex interactions between different prognostic factors. Our approach represents an inverse paradigm in cancer risk stratification through enabling the discovery of prognostically relevant patterns directly from patient data without prior assumptions or feature selection. This data-driven approach has the potential to identify novel risk factors and patient subgroups that might be missed by conventional staging systems^44,64,65^. This flexibility is particularly valuable in the era of precision medicine, where treatment decisions increasingly depend on integrating diverse data types to characterize individual patients’ disease biology^69^.

Here, our analysis of bone marrow plasma cells’ transcriptomics sought to demonstrate a potential application-case of our proposed method and how it may be employed to elucidate determinants of differential prognosis in contexts where underlying mechanisms are not yet fully understood. However, it should be noted that the presented results in the context of transcriptomic data are far from comprehensive. While we employed conscientious cross-validation throughout our analysis, thereby precluding overfitting of our model, these results should be understood as merely exploratory since they do not necessarily reflect biological causalities. The identified transcripts and their relationships to survival outcomes should therefore be viewed primarily as hypothesis-generating and may lay the groundwork for further bioinformatical characterization. Further studies with independent cohorts and functional biological analyses would be required to confirm the clinical and biological significance of these findings.

Still, the decent separation of the identified subgroups and the identification of novel transcripts driving cluster assignments goes to show how our method may help uncover new actionable clinical insights and shape the understanding of disease biology.

As we continue to refine and expand this methodology, we envision its integration into clinical decision support systems that can help oncologists navigate the growing complexity of cancer diagnosis, prognosis, and treatment selection. Before clinical deployment, however, a necessary requirement would be to validate the safety of our method for specific applications through prospective clinical trials demonstrating that decisions guided by our risk stratification lead to improved patient outcomes compared to current standard-of-care approaches. Additionally, regulatory approval processes and integration with existing electronic health record systems would be essential steps to enable seamless integration with clinical workflows. Upon successful completion of these validation steps, clinicians would ideally interact with such a tool by inputting routine clinical data (laboratory values, imaging studies) to receive automated risk stratification alongside transparent feature importance rankings, enabling more personalized treatment decisions while maintaining a human-centric approach that complements rather than replaces critical clinical judgment. Beyond clinical application, our tool promises to also serve as a research accelerator, drawing clinical researchers’ attention towards factors driving differential prognosis in contexts where understanding of these factors remains limited. This could prove particularly valuable in emerging therapeutic areas like CAR T-cell therapy in MM where risk stratification approaches continue to be refined^51,52^ but are still in their infancy, promising to be a fruitful avenue for future research.

In conclusion, our study introduces a novel optimization framework for training neural networks to identify prognostically distinct patient subgroups across diverse cancer types and data modalities, through direct optimization of survival heterogeneity. Our approach enables the discovery of clinically interpretable patient stratifications without prior knowledge of established risk factors or extensive manual feature engineering. Thus, by providing a unified approach to patient stratification across different cancer types and data modalities, our framework contributes to the broader goal of precision oncology: matching patients with the most effective treatments at the right time^23,70^.

## Methods

### Ethics statement

The CoMMpass study was funded by the MM Research Foundation (MMRF) and conducted in line with the Declaration of Helsinki. Approval for the study was granted to the MMRF by the second panel of the Western Institutional Review Board. The CoMMpass study data is publicly deposited by the MMRF in anonymized form (refer to section *Data availability*). Participants were not paid for their involvement in the CoMMpass study and only joined the study after giving their written informed consent.

The GMMG-MM5 trial was carried out in compliance with the European Clinical Trial Directive (2005) and the Declaration of Helsinki. The GMMG received ethical approval from the University of Heidelberg’s ethics committee. Participants were not paid for their involvement in the MM5 study and only joined the study after giving their written informed consent. The MM5 study data was shared with the University of Leipzig in anonymized form and was processed in a GDPR-compliant manner.

The Lung1 study was conducted according to Dutch law and in line with the Declaration of Helsinki. Approval for the Lung1 study was granted to Aerts et al.^32^ by the local ethics committee at Maastricht University Medical Center (MUMC1), Maastricht, The Netherlands. The Lung1 study data is publicly deposited by Aerts et al. at The *Cancer Imaging Archive* in anonymized form (refer to section *Data availability*).

NSCLC patients treated at the University Hospital Leipzig gave written informed consent for use of their data and were not paid for their involvement in this study.

All research in our study was carried out retrospectively with the publicly available data and in compliance with the Declaration of Helsinki.

### Patient characteristics and data preprocessing

For model training and validation on clinical tabular data, we retrieved MM laboratory patient data from the CoMMpass study (NCT01454297) database version IA21. For MM disease kinetics modeling, we adopted the parameter selection from our previous work^71^, which identified key biomarkers based on their clinical relevance: Hb (anemia prevalence), Calcium (Ca) (bone metabolism and hypercalcemia), Creatinine (Cr) (renal complications), White blood bells (WBC) (treatment-related leukopenia and infection risk), MM-specific disease markers (M-Protein (M-Pr), SFL), and prognostic indicators (LDH, Alb, *β*2m). Furthermore, we have chosen these parameters because they represent standard laboratory measurements in MM care and are therefore easily accessible in routine clinical practice. To assess model performance, we employed a 5-fold cross validation scheme, where we trained the model on 80% of patients and evaluated its performance on the remaining 20% unseen patients across five different partitions of the data. The only patients excluded were those for which no survival documentation was available, which left 998 patients in total, resulting to 799 patients for model training and 199 patients for validation in each of the five cross validation folds respectively. Missing values for lab values where imputed using the mean value of each respective parameter. Importantly, imputation parameters were derived from the training data exclusively to avoid bias in the evaluation of model performance. Most importantly, we only imputed the input features of our models, which enabled the predictition of groups assignemnts for incomplete data. Critically, we did not impute any outcome parameters that were used to evaluate model performances. All laboratory parameters were subject to a feature-wise standard normalization (mean = 0, standard deviation = 1). Likewise, normalization parameters were derived from the training data exclusively to avoid bias. Table 3 conveys an overview of the relevant patient characteristics.

**Table 3:** Patient characteristics table for MM cohort separated by cross-validation (*k*^*th*^) fold

	*k*=1 (*N*=199)	*k*=2 (*N*=199)	*k*=3 (*N*=199)	*k*=4 (*N*=199)	*k*=5 (*N*=199)
Age (years)	64.8 ± 11.6	63.6 ± 10.5	64.5 ± 10.5	63.0 ± 9.7	63.4 ± 10.8
**ISS** **Stage**					
ISS 1	65 (32.7%)	75 (37.7%)	67 (33.7%)	63 (31.7%)	62 (31.2%)
ISS 2	71 (35.7%)	59 (29.6%)	60 (30.2%)	69 (34.7%)	87 (43.7%)
ISS 3	56 (28.1%)	56 (28.1%)	64 (32.2%)	62 (31.2%)	49 (24.6%)
**Stem Cell Transplant (SCT)**					
No SCT	98 (49.2%)	101 (50.8%)	96 (48.2%)	77 (38.7%)	98 (49.2%)
SCT	101 (50.8%)	98 (49.2%)	103 (51.8%)	122 (61.3%)	101 (50.8%)
**Treatment Class**					
Bortezomib-based	49 (24.6%)	39 (19.6%)	52 (26.1%)	31 (15.6%)	43 (21.6%)
Carfilzomib-based	1 (0.5%)	2 (1.0%)	6 (3.0%)	6 (3.0%)	0 (0.0%)
IMIDs-based	11 (5.5%)	15 (7.5%)	8 (4.0%)	16 (8.0%)	16 (8.0%)
Combined IMIDs/carfilzomib-based	18 (9.0%)	14 (7.0%)	11 (5.5%)	19 (9.5%)	13 (6.5%)
Combined bortezomib/IMIDs-based	115 (57.8%)	121 (60.8%)	114 (57.3%)	114 (57.3%)	115 (57.8%)
Combined bortezomib/IMIDs/carfilzomib-based	5 (2.5%)	7 (3.5%)	8 (4.0%)	12 (6.0%)	11 (5.5%)
Combined bortezomib/carfilzomib-based	0 (0.0%)	0 (0.0%)	0 (0.0%)	1 (0.5%)	1 (0.5%)
Combined daratumumab/IMIDs/carfilzomib-based	0 (0.0%)	1 (0.5%)	0 (0.0%)	0 (0.0%)	0 (0.0%)
**Laboratory Values**					
Hemoglobin [mmol/L]	6.64 ± 1.21	6.79 ± 1.13	6.64 ± 1.22	6.61 ± 1.17	6.63 ± 1.24
Calcium [mmol/L]	2.35 ± 0.25	2.38 ± 0.31	2.42 ± 0.29	2.39 ± 0.33	2.35 ± 0.30
Creatinine [*μ*mol/L]	119.61 ± 111.17	129.38 ± 120.74	125.50 ± 94.44	131.93 ± 172.66	114.64 ± 98.38
LDH [*μ*kat/L]	3.40 ± 1.79	3.68 ± 2.43	3.47 ± 1.96	3.51 ± 2.92	3.29 ± 1.95
Albumin [g/L]	35.32 ± 6.42	36.10 ± 6.45	37.12 ± 6.22	34.94 ± 6.31	35.62 ± 6.20
*β*-2-Microglobulin [mg/L]	5.20 ± 4.80	4.96 ± 4.47	5.45 ± 4.99	5.39 ± 5.10	4.88 ± 4.03
M-protein [g/dL]	2.63 ± 2.17	2.68 ± 2.38	2.77 ± 2.39	2.98 ± 2.30	2.80 ± 2.37
Lambda light chain [mg/dL]	130.04 ± 830.95	119.23 ± 516.70	96.50 ± 322.00	126.32 ± 596.15	87.39 ± 268.72
Kappa light chain [mg/dL]	315.85 ± 1810.64	127.19 ± 291.28	244.61 ± 819.26	216.74 ± 710.28	2743.83 ± 32459.86
White blood cell count [10^9^/L]	6.11 ± 2.35	6.53 ± 2.93	6.25 ± 2.20	6.24 ± 2.42	6.50 ± 3.33
**Survival Outcomes**					
Median survival [days (IQR)]	1749 (632—2652)	1770 (610—2513)	1986 (780—2580)	1944 (796—2559)	1690 (789—2512)
Censoring rate	54.3%	60.8%	58.3%	61.8%	53.3%

We used data from the GMMG-MM5 phase III trial conducted by the GMMG (EudraCT No. 2010-019173-16)^27,72,73^ for external validation of the MM model constructed using our method. For a comprehensive description of patient and disease characteristics, as well as the study design, we refer the reader to the original publication by Mai et al.^27^.

For model training and validation on clinical imaging data, we retrieved CT scans of NSCLC patients from the Lung1 study dataset version 4 (2020)^31^. The Lung1 study dataset includes 422 patients with documented clinical outcomes and is publicly available at the *Cancer Imaging Archive*. The inclusion criteria for the study were a confirmed primary tumour in the CT scan and curative-intent treatment^32^. The unaltered CT scans were normalized for model training using zero-signal preserving contrast enhancement. This normalization subjects tissue pixel intensities to a standard normalization (mean = 0, standard deviation = 1), while background pixels remain unchanged, which effectively makes subtle structural differences more detectable by amplifying intensity variations within tissue regions. Like in the application to MM, model performance was assessed using a 5-fold cross-validation scheme, where we trained the model on 80% of patients and evaluated its performance on the remaining 20% unseen patients across five different partitions of the data. We did not exclude any patients from the dataset in our analysis, which resulted in 336 patients for training and 84 patients for validation in each of the five cross validation folds. Furthermore, the training data was augmented by decomposing 3D CT scans into 2D axial slices, selecting 2 to 3 slices per patient that contained visible NSCLC tissue, yielding a total of 1118 slices for model training. Our choice of 2D slice analysis was motivated by evidence from the literature demonstrating that 2D radiomics signatures can match or even outperform 3D radiomics signatures in CT scans of NSCLC^74^, while allowing us to maintain a CNN architecture with a manageable parameter space (as compared to 3D CNNs) appropriate for our dataset size. Validation was done on a single slice per patient which contained the largest cross-section of NSCLC tissue. Table 4 conveys an overview of the relevant patient characteristics.

**Table 4:** Patient characteristics table for NSCLC cohort separated by cross-validation (*k*^*th*^) fold

	*k*=1 (*N*=84)	*k*=2 (*N*=84)	*k*=3 (*N*=84)	*k*=4 (*N*=84)	*k*=5 (*N*=84)
Age (years)	68.3 ± 10.0	69.2 ± 9.6	68.3 ± 10.1	67.8 ± 10.1	66.6 ± 10.6
**T** **Stage**					
T1	16 (19.0%)	17 (20.2%)	25 (29.8%)	21 (25.0%)	14 (16.9%)
T2	31 (36.9%)	29 (34.5%)	30 (35.7%)	31 (36.9%)	35 (42.2%)
T3	13 (15.5%)	12 (14.3%)	8 (9.5%)	9 (10.7%)	10 (12.0%)
T4	24 (28.6%)	24 (28.6%)	21 (25.0%)	23 (27.4%)	24 (28.9%)
T5	0 (0.0%)	2 (2.4%)	0 (0.0%)	0 (0.0%)	0 (0.0%)
**N** **Stage**					
N0	38 (45.2%)	27 (32.1%)	38 (45.2%)	37 (44.0%)	29 (34.5%)
N1	5 (6.0%)	5 (6.0%)	7 (8.3%)	3 (3.6%)	2 (2.4%)
N2	28 (33.3%)	31 (36.9%)	22 (26.2%)	31 (36.9%)	29 (34.5%)
N3	13 (15.5%)	20 (23.8%)	16 (19.0%)	13 (15.5%)	23 (27.4%)
**M** **Stage**					
M0	84 (100.0%)	82 (97.6%)	83 (98.8%)	84 (100.0%)	82 (97.6%)
M1	0 (0.0%)	1 (1.2%)	0 (0.0%)	0 (0.0%)	0 (0.0%)
M2	0 (0.0%)	0 (0.0%)	0 (0.0%)	0 (0.0%)	0 (0.0%)
M3	0 (0.0%)	1 (1.2%)	1 (1.2%)	0 (0.0%)	2 (2.4%)
**Overall** **Stage**					
I	18 (21.4%)	13 (15.7%)	24 (28.6%)	23 (27.4%)	15 (17.9%)
II	10 (11.9%)	11 (13.3%)	9 (10.7%)	3 (3.6%)	7 (8.3%)
IIIa	20 (23.8%)	24 (28.9%)	17 (20.2%)	29 (34.5%)	21 (25.0%)
IIIb	36 (42.9%)	35 (42.2%)	34 (40.5%)	29 (34.5%)	41 (48.8%)
**Histology**					
Adenocarcinoma	11 (14.7%)	11 (14.5%)	9 (12.3%)	7 (9.5%)	13 (16.2%)
Large cell	20 (26.7%)	22 (28.9%)	30 (41.1%)	22 (29.7%)	20 (25.0%)
NOS	15 (20.0%)	14 (18.4%)	5 (6.8%)	10 (13.5%)	17 (21.2%)
Squamous cell carcinoma	29 (38.7%)	29 (38.2%)	29 (39.7%)	35 (47.3%)	30 (37.5%)
**Survival** **Outcomes**					
Median survival [days (IQR)]	516 (248—1477)	563 (286—1411)	563 (260—1526)	528 (280—1034)	675 (277—1510)
Censoring rate	10.7%	11.9%	13.1%	8.3%	13.1%

CT imaging of the Leipzig NSCLC patients was performed in a clinical setting with a 256 slice CT scanner (iCT, Philips, Amsterdam, Netherlands) after intravenous application of 90 mL iodinated intravenous contrast medium (injected at a rate of 2 mL/s by a power injector, Medtron GmbH, Germany), with a scan delay of 70 seconds after onset of injection for portal-venous phase. Imaging parameters were 120 kVp and 150-300 mAs. Reconstructed slice thickness was 1 mm. From the CT data, we extracted axial slices containing visible NSCLC tissue. Chosen slices were thresholded using Otsu’s method^75^ to remove background, followed by zero-padding to match image dimensions of the Lung1 dataset.

### Development of a differentiable optimization criterion

Consider a survival analysis setting with *k* distinct groups comprising *n* individuals. Initially, let *g*_*i*_ ∈ {1, 2, 3, . . . , *k*} represent the definitive group membership of the *i*-th individual. The Multivariate Logrank statistic *L*, which quantifies the heterogeneity of survival curves across groups, can be constructed as follows: For each group *g* and unique event time *t*_*j*_, the observed events *O*_*g*,*j*_ are calculated using:

3 $${O}_{g,j}=\mathop{\sum }\limits_{i\in D({t}_{j})}{\delta }_{{g}_{i},g}\,\,{\rm{with}}\,\,{\delta }_{{g}_{i},g}=\left\{\begin{array}{ll}1 & {\rm{if}}\,{g}_{i}=g\\ 0 & {\rm{if}}\,{g}_{i}\ne g\end{array}\right.$$

where *D*(*t*_*j*_) represents the set of individuals experiencing the event at time *t*_*j*_, and ${\delta }_{{g}_{i},g}$ indicates group membership. The corresponding expected events *E*_*g*,*j*_ for group *g* at time *t*_*j*_ are given by:

4 $${E}_{g,j}=\frac{{R}_{g}({t}_{j})}{| R({t}_{j})| }\cdot {d}_{j}$$

Here, *R*_*g*_(*t*_*j*_) denotes the number of at-risk individuals in group *g* at time *t*_*j*_, ∣*R*(*t*_*j*_)∣ represents the total at-risk population across all groups at time *t*_*j*_, and *d*_*j*_ accounts for the total number of events at time *t*_*j*_, incorporating censoring information. The multivariate log-rank statistic is then:

5 $$L={{\bf{Z}}}^{T}{{\bf{V}}}^{-1}{\bf{Z}}$$

where ${\bf{Z}}={[{Z}_{1},{Z}_{2},...,{Z}_{k}]}^{T}$ with *Z*_*g*_ = ∑_*j*_(*O*_*g*,*j*_ − *E*_*g*,*j*_) and **V** represents the full variance-covariance matrix that accounts for correlations between groups, calculated using the standard hypergeometric variance formula for the log-rank test^76,77^.

To leverage modern machine learning based clustering techniques, we can generalize this framework to accommodate soft class assignments. This is achieved by redefining *g*_*i*_ as a categorical probability distribution over the class labels:

6 $${g}_{i}=[{p}_{i1},{p}_{i2},\ldots ,{p}_{ik}]\,\,\mathrm{with}\,\,\int {g}_{i}=1$$

where *p*_*ig*_ reflects the probability of individual *i* to belong to group *g*. Here, *g*_*i*_ is modeled as a function ${g}_{i}({\vec{x}}_{i},\theta )$ of the features ${\vec{x}}_{i}$ of individual *i* and model parameters *θ* of a neural network. This probabilistic framework generalizes the observed events calculation to partial events:

7 $${O}_{g,j}=\mathop{\sum }\limits_{i\in D({t}_{j})}{p}_{ig}$$

where *D*(*t*_*j*_) remains the total set of individuals experiencing the event at time *t*_*j*_. Similarly, the risk set for group *g* at time *t*_*j*_ becomes a weighted sum of probabilities:

8 $${R}_{g}({t}_{j})=\mathop{\sum }\limits_{i\in R({t}_{j})}{p}_{ig}$$

where *R*(*t*_*j*_) is then a set of partial individuals at risk at time *t*_*j*_ and allows for calculation of expected events *E*_*g*,*j*_ for group *g* at time *t*_*j*_ identically to Eqn. (4).

The variance-covariance matrix **V** and the observed minus expected events **Z** also extend naturally to the probabilistic setting, so that the log-rank loss *L* can be computed identically to Eqn. (5) based on the evaluation of Eqn. (7), Eqn. (8), and Eqn. (4). This formulation preserves the statistical properties of the log-rank statistic while allowing for soft class assignments. Naturally, the assumptions underlying the original log-rank statistic (proportional hazards, non-informative censoring, etc.) similarly apply to this loss function implementation. The advantage of this probabilistic formulation is that it creates a fully differentiable objective function, which enables gradient-based optimization of the model parameters *θ* through differentiation of *L* with respect to *θ* using the chain rule:

9 $$\frac{\partial L}{\partial \theta }=\mathop{\sum }\limits_{g}\left(\frac{\partial L}{\partial {O}_{g}}\frac{\partial {O}_{g}}{\partial g}\frac{\partial g}{\partial \theta }+\frac{\partial L}{\partial {E}_{g}}\frac{\partial {E}_{g}}{\partial g}\frac{\partial g}{\partial \theta }+\frac{\partial L}{\partial V}\frac{\partial V}{\partial g}\frac{\partial g}{\partial \theta }\right)$$

so that the model parameters *θ* can then be optimized iteratively based on the gradient of *L* by applying established learning rules, i.e.:

10 $$\theta \leftarrow \theta +\alpha \frac{\partial L}{\partial \theta }+R(\theta )$$

As a result, changes in *θ* affect the predicted probabilities *p*_*ig*_, which in turn propagate through the observed events, expected events, and variance calculations to the final log-rank loss. By virtue of this approach, our method enables training models to map patient features to cluster assignments, allowing direct optimization of distinct patient groups with maximally different survival outcomes.

### Model implementation and training

All employed models were implemented using the PyTorch^24^ framework (version 2.5.1) and trained using PyTorchLightning^78^ (version 2.5.0.post0). The MLP for application on real-world clinical biomarker data of MM patients was configured as follows:

$$\begin{array}{rcl} & & Linear(10\mapsto 256)+ReLU\\ & & Linear(256\mapsto 256)+ReLU\\ & & Linear(256\mapsto 256)+ReLU\\ & & Linear(256\mapsto 256)+ReLU\\ & & Linear(256\mapsto 256)+ReLU\\ & & Linear(256\mapsto 3)+Softmax.\end{array}$$

The model was trained at a learning rate of 10^−3.9^, for 20 epochs with a mini-batch size of 128, using a uniform weight-decay of 10^−2.2^ applied to weights and biases. The class-imbalance penalty for our custom *PartialMultivariateLogRankLoss* was tuned to 10^−0.7^ The MM-MLP model architecture as well as the training hyperparameterization was obtained via Bayesian optimization using a Gaussian process sampler from optuna^29^ (version 4.6.0).

The NSC model was trained without architectural modifications and its hyperparameterization was tuned analogously to the MLP. The found parameters were:

- survival layer hidden size: 128
- representation layer hidden size: 32
- arbitrary hidden layer size: 128
- number of hidden layers: 1 (per module)
- learning rate: 10^−3^
- mini-batch size: 64

The CNN for application on real-world CT imaging data of (NSCLC) patients was configured with six convolution-and-pooling blocks followed by a single linear layer with a Softmax activation:

$$\begin{array}{rcl} & & Convolution(1\mapsto 32,\,16\times 16)+MaxPooling(4\times 4)\\ & & Convolution(32\mapsto 8,\,8\times 8)+MaxPooling(4\times 4)\\ & & Convolution(8\mapsto 8,\,8\times 8)+MaxPooling(4\times 4)\\ & & Convolution(8\mapsto 8,\,8\times 8)+MaxPooling(2\times 2)\\ & & Convolution(8\mapsto 4,\,3\times 3)+MaxPooling(2\times 2)\\ & & Convolution(4\mapsto 4,\,2\times 2)+MaxPooling(2\times 2)\\ & & Linear(4\mapsto 2)+Softmax.\end{array}$$

All convolutional layers use zero-padding. Through the successive convolution and pooling operations, the spatial dimensions (height and width) of the feature maps are progressively reduced until the final 4-channel activation map comprises a one-dimensional vector that can be directly passed to the fully-connected layer. The model was trained at a learning rate of 10^−3^ for 100 epochs with mini-batches of 32 images, using a uniform weight-decay of 10^−1^ applied to weights and biases. The class-imbalance penalty of our custom *PartialMultivariateLogRankLoss* was set to 10^−1^, Model hyperparameters and training parameters were determined through manual grid search.

All models were trained employing an AdamW optimizer^79^. We selected AdamW, a variation of the Adam optimizer, due to its adaptive learning rate capabilities and decoupled weight decay regularization, which was shown to outperform more simplistic optimizers, such as stochastic gradient descent, across most machine learning applications^80^.

### Statistics

We used nonparametric bootstrap resampling to quantify variability in C-indices reported in Table 2 and to test pairwise differences in performance^81,82^. For estimation of C-index variability, the respective cohorts were resampled with replacement 9,999 times; for each bootstrap sample we computed the C-index on the relevant validation data (internal cross-validation for CoMMpass or external validation for GMMG-MM5). The reported C-index summary for each model is the median of the 9999 bootstrap estimates, and 95% confidence intervals, taken from the 2.5th and 97.5th percentiles of the bootstrap distribution.

For pairwise comparisons between the MLP and each comparator we performed hypothesis testing based on the nonparametric bootstrap distribution of paired differences in model performance. To this end, we computed the C-index for the MLP and for the comparator on the same bootstrap sample of the cohort, recorded the paired difference Δ, and constructed the bootstrap distribution of these paired differences using 9999 bootstrap samples. We then derived one-sided p-values by computing the percentile position of zero in the paired-difference distribution (i.e., min(p(Δ ≤ 0), p(Δ ≥ 0)))^81,82^. For pairwise comparisons between the MLP and each respective estimator, only validation data was subjected to bootstrapping, resulting in performance evaluation, which is based entirely on unseen patients by either estimator.

### Cytogenetic classification criteria

Chromosomal abberations (CA) in MM were identified using whole genome sequencing (WGS) data available for the CoMMpass cohort. Del(17p) only was considered when mutation was exclusively detectable in del(17p) measurement, while mutation only required detectability in both del(17p) and TP53 assays. Translocations t(4;14) and t(14;16) were considered positive based on calls detected in *NSD2* and *MAF*/*MAFA* genes, respectively. Based on the established classification crtiteria^10^, CA were classified as high-risk if the presence of either del(17p) and/or t(4;14) and/or t(14;16) was detected. All other cases were classified as negative for the respective alterations.

### SHAP value computation

To approximate feature contributions for the outputs of our models, we calculated SHAP values analogous to our previous work^71^. SHAP values are an estimation of classic Shapley values from game theory^83^, which in the context of machine learning allows approximating each feature’s contribution to a given model’s predictions. In brief, we used the DeepExplainer class^84,85^ of the SHAP python library^86^ (version 0.46.0). In a given cross-validation fold, the DeepExplainer was provided with the entire training partition as background datasets and the entire testing partition as the input (refer to methods section *Patient characteristics and data preprocessing* for details about data partitioning). The SHAP values were computed for each cross-validation fold individually and then combined in the final analysis.

## Supplementary information

Supplementary Information

## Data Availability

The CoMMpass data is available upon registration in the Multiple Myeloma Research Foundation (MMRF) Researcher Gateway at https://research.themmrf.org. Inquiries about the GMMG-MM5 study data should be addressed to hartmut.goldschmidt@med.uni-heidelberg.de. The Lung1 data was originally part of a study by Aerts et al.^32^ and is publicly available at the *Cancer Imaging Archive*^31^. Inquiries about the Leipzig NSCLC data should be addressed to the corresponding author.

## Code availability

We hosted a public GitHub repository at https://github.com/maximilianferle/Unsupervised-risk-factor-identification-across-cancer-types-and-data-modalities-via-explainable-AI with all code used to produce the results of this study.
