# Publisher Correction: Unsupervised risk factor identification across cancer types and data modalities via explainable artificial intelligence

**DOI:** 10.1038/s41746-026-02833-w

**Published:** 2026-06-16

**Authors:** Maximilian Ferle, Jonas Ader, Thomas Wiemers, Nora Grieb, Beatrice Berneck, Adrian Lindenmeyer, Hartmut Goldschmidt, Elias K. Mai, Uta Bertsch, Hans-Jonas Meyer, Thomas Neumuth, Markus Kreuz, Kristin Reiche, Maximilian Merz

**Affiliations:** 1 Center for Scalable Data Analytics and Artificial Intelligence (ScaDS.AI) Dresden/Leipzig, Universität Leipzig, Leipzig, Germany; 2 Innovation Center Computer Assisted Surgery (ICCAS), University of Leipzig, Leipzig, Germany; 3 Department of Diagnostics, Fraunhofer Institute for Cell Therapy and Immunology, Leipzig, Germany; 4 Department of Hematology, Hemostaseology, Cellular Therapy and Infectiology, University Hospital of Leipzig, Leipzig, Germany; 5 Department of Internal Medicine V, University Hospital Heidelberg, Heidelberg, Germany; 6 National Center for Tumor Diseases (NCT), Heidelberg, Germany; 7 Department of Diagnostic and Interventional Radiology, University Hospital Leipzig, Leipzig, Germany; 8 Institute for Clinical Immunology, University Hospital of Leipzig, Leipzig, Germany; 9 Myeloma Service, Memorial Sloan Kettering Cancer Center, New York, NY USA

**Keywords:** Cancer, Computational biology and bioinformatics, Oncology

Correction to: *npj Digital Medicine* 10.1038/s41746-026-02663-w, published online 11 May 2026

The original version of this article contained typographical errors in the axis labels of Figure 2b, Figure 2e, and Figure 3b. The original article has been corrected.

