# Supplementary material for: Unsupervised risk factor identification across cancer types and data modalities via explainable artificial intelligence
Source: NPJ Digit Med. 2026 May 11;9:363. doi: 10.1038/s41746-026-02663-w (PMC13161318; doi:10.1038/s41746-026-02663-w)
Supplement: Supplementary file 1 — Supplementary Information [file 41746_2026_2663_MOESM1_ESM.pdf]

Supplementary Results

Performance assessment of our method on simulated data

To validate our method in the common clinical scenario of analyzing structured patient data (Supplementary Figure 1a), we generated synthetic datasets comprising three-dimensional feature vectors, where each dimension represented a continuous variable analogous to laboratory parameters or clinical measurements commonly encountered in medical practice.

The controlled simulation experiment addresses the challenge of survival-based clustering without knowledge of a definitive ground truth and allowed us to evaluate our method's ability to recover predefined relationships between feature distributions and survival outcomes.

To this end, we defined three distinct ground truth groups by sampling feature vectors from overlapping multivariate normal distributions (Supplementary Figure 1b). This approach models the inherent biological variability and partial overlap of biomarker profiles observed in clinical populations, thus creating a challenging yet realistic scenario for testing our clustering algorithm's discriminative capabilities.

For each ground truth group, we sampled survival times from group-specific Weibull distributions (Supplementary Figure 1c). We selected Weibull distributions as it is a common choice in modeling survival distributions due to their flexibility in modeling various hazard patterns commonly observed in clinical survival data[87,88].

The Weibull distribution was selected due to its flexibility in modeling various hazard functions commonly observed in medical data[87,88]. Event times were generated using inverse-transform sampling from the Weibull distribution:

$$T = \lambda(-\ln(1-U))^{1/\rho}, \qquad \text{(Supplementary Eqn. 1)}$$

where U follows a uniform distribution on $[0, 1]$. Group-specific parameters were:

$$\begin{aligned} \text{Group 0: } & \rho_0 = 0.539, \lambda_0 = 3068.812, \\ \text{Group 1: } & \rho_1 = 0.898, \lambda_1 = 5114.687, \\ \text{and Group 2: } & \rho_2 = 1.257, \lambda_2 = 7160.562, \end{aligned}$$

where ρ represents the shape parameter and λ the scale parameter. The parameters were obtained by using the CoMMpass dataset as a reference, stratifying patients by the ISS stage[45], and fitting Weibull distributions to each group. Through this process, we obtained a set of three group-specific survival distributions that were based on real-world evidence, which accurately reflected a clinical prognostic setting. To simulate realistic clinical scenarios with censoring, we introduced random censoring using exponential distribution with scale parameter $10,000$ to mirror censoring rates typical of clinical studies. Additionally, to create the characteristic incomplete follow-up pattern common in survival analysis, we introduced random right-censoring by generating competing censoring times from an exponential distribution. Administrative censoring was applied at $4,000$ time units to simulate finite study follow-up periods. The Kaplan-Meier curves resulting from sampling survival times for these ground truth groups (Supplementary Figure 1d) demonstrate clear separation.

We implemented a Multilayer Perceptron (MLP) architecture (Supplementary Figure 1a) to map the three-dimensional feature vectors to group assignments.

The MLP for clustering the synthetic feature vectors associated with survival data was configured with one hidden layer and an output layer:

$$\begin{aligned} &\mathit{Linear}\ 3 \mapsto 16 \\ &\mathit{Linear}\ 16 \mapsto 3 + \mathit{Softmax}. \end{aligned}$$

The model was trained at a learning rate of 0.01, for 50 epochs with a mini-batch size of 32, using a uniform weight-decay of 0.01 applied to weights and biases.

Critically, the MLP was trained exclusively using our custom *PartialMultivariateLogrankLoss* function, without any knowledge of the predefined group assignments and learned survival-relevant patterns solely through the optimization of between-group survival heterogeneity using our custom *PartialMultivariateLogrankLoss* function.

The MLP demonstrated robust performance in recovering the underlying survival patterns from the synthetic tabular data (Table 1). Quantitative assessment through ROC analysis (Table 1, Supplementary Figure 1d) suggests strong discriminative performance, achieving AUROC values of 0.96 ± 0.00, 0.96 ± 0.01, and 0.96 ± 0.00 for Classes 0, 1, and 2, respectively. The precision-recall curve (PRC) analysis (Table 1, Supplementary Figure 1e) also showed strong performance, with AUPRC values of 0.92 ± 0.01, 0.92 ± 0.01, and 0.93 ± 0.01 for Classes 0, 1, and 2, respectively, indicating high precision of the model's predictions across all three classes. These values indicate that by training the model to optimize between-group survival heterogeneity using our custom *MultivariateLogrankLoss*, the MLP can effectively distinguish between the three risk groups despite the challenge of overlapping feature distributions and censored survival data.

Comparison of the ground truth Kaplan-Meier survival curves (Supplementary Figure 1f) with those identified by the model (Supplementary Figure 1g) indicates that the model successfully reproduced the distinct survival trajectories of each group.

Finally, we investigated the impact of variations in model parameters k and penalty weight λ across different numbers of ground truth groups g on a given model's predictions. To this end, we compared the entropy of predicted class assignments made by a model when trained on synthetic tabular data with the beforementioned configurations (Supplementary Figure 2). Conceptually, larger entropy reflects the increased disorder of the predictive distribution, thereby providing a readout of the informativeness of class assignments. For each combination (g, k) we trained models with penalty weight $\lambda \in \{0.1, 0.5, 0.8, 1.1, 1.5, 2.0\}$ and computed the mean entropy of the models' predictions, normalized by the maximum possible entropy given k. Here we would observe that at relatively low values of λ, predicted class assignments were uniformly informative across configurations, with entropies continually rising until finally approaching the k-specific maximum for sufficiently large λ (Supplementary Figure 2a-f). As expected, stronger class-imbalance penalization pushes predicted class frequencies toward uniformity, thereby increasing entropy making class assignments less informative, while for appropriate values of λ the models would successfully converge to a stable state where predictions were informative across configurations.

To further demonstrate the versatility of our approach across data modalities and model architectures, we extended our validation to the domain of computer vision. To simulate the clinical setting of pattern recognition in heterogeneous imaging data, we used the MNIST dataset of handwritten digits[89,90] from the UCI Machine Learning Repository (`https://archive.ics.uci.edu`), which contains 8×8 pixel grayscale images of handwritten digits 0-9. While maintaining the same core optimization framework based on our *PartialMultivariateLogrankLoss*, we transitioned from a MLP architecture to a Convolutional Neural Network (CNN) suited for image processing. Again, we used a controlled simulation experiment to evaluate the performance of our method to overcome the absence of ground truth for optimal survival-based clustering in real-world imaging data.

We excluded digit 0 and retained digits 1-9 and normalized each image using standard scaling to ensure consistent feature magnitudes across the dataset. Based on digit labels we defined a mapping to one of the three risk groups in a randomized fashion using a fixed permutation (Supplementary Figure 3b). The final dataset comprised of approximately $5,000$ samples with known group memberships and distinct survival and censoring distributions for each group. All random number generation was performed with fixed seeds to ensure reproducibility.

Here, we parameterized our experiment analogous to before by associating specific handwritten digits with distinct survival distributions. To this end, we simulated survival times by sampling from group-specific Weibull distributions (Supplementary Figure 3c) accounting for incomplete follow-ups analogous to our previous experiment for each of the three pre-defined groups. By doing so, we could simulate the clinical situation of imaging data being associated with different outcomes and measure our method's ability to recover these predefined relationships.

We implemented a CNN (Supplementary Figure 3a) to process the digit images and map them to group assignments.

The CNN was configured with three convolution-and-pooling blocks followed by a single linear layer with a Softmax activation:

$$\begin{aligned}
&\textit{Convolution } 1 \mapsto 64\ (5\times 5) + \textit{MaxPooling } (2\times 2)\\
&\textit{Convolution } 64 \mapsto 32\ (3\times 3) + \textit{MaxPooling } (2\times 2)\\
&\textit{Convolution } 32 \mapsto 8\ (5\times 5) + \textit{MaxPooling } (2\times 2)\\
&\textit{Linear } 8 \mapsto 3 + \textit{Softmax}.
\end{aligned}$$

All convolutional layers use zero-padding. Through the successive convolution and pooling operations, the spatial dimensions (height and width) of the feature maps are progressively reduced until the final 8-channel activation map comprises a one-dimensional vector that can be directly passed to the fully-connected layer. The model was trained at a learning rate of 0.001, for 20 epochs with a mini-batch size of 64, using a uniform weight-decay of 0.01 applied to weights and biases.

Critically, as with our previous MLP experiment, the CNN was trained exclusively using our *PartialMultivariateLogrankLoss* function, without any knowledge of the predefined group assignments or digit identities. The model learned to identify survival-relevant patterns solely through optimization of between-group survival heterogeneity.

The CNN demonstrated excellent performance in recovering the underlying survival patterns from the image data (Table 1). Quantitative assessment through ROC analysis (Table 1, Supplementary Figure 3d) suggested strong strong discriminative performance, achieving AUC values of 0.97 ± 0.00, 0.94 ± 0.01, and 0.98 ± 0.00 for Classes 0, 1, and 2, respectively. The PRC analysis (Table 1, Supplementary Figure 1e) also showed strong performance, with AUPRC values of 0.94 ± 0.01, 0.91 ± 0.01, and 0.95 ± 0.01 for Classes 0, 1, and 2, respectively, indicating high precision of the models' predictions across all three classes. These values indicate that our method enabled the CNN to effectively distinguish between the three groups solely based on associating the image data with survival times. Comparison of the ground truth

Kaplan-Meier survival curves (Supplementary Figure 3f) with those identified by the model (Supplementary Figure 3g) reveals that the model successfully reproduced the distinct survival trajectories of each group.

The successful application of our methodology to this computer vision task using the same core optimization approach highlights its robustness across different network architectures and data modalities.

Encouraged by these strong results in controlled settings, we extended our analyses to applying our framework to a set of challenging real-world clinical problems.

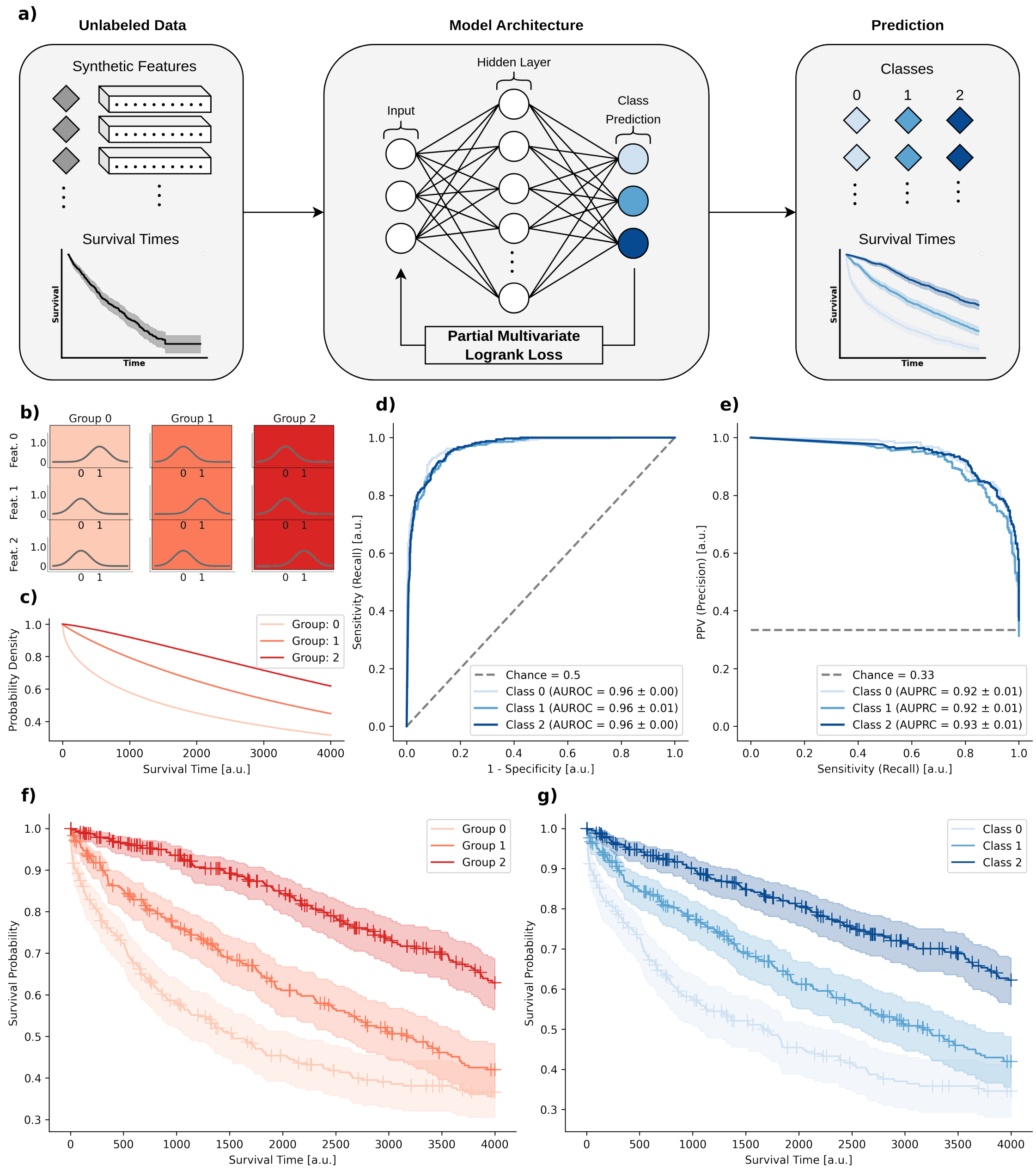

Supplementary Figure 1: **Stratification of synthetic feature vectors according to their associated survival distributions. (a)** Architecture diagram showing the multilayer perceptron workflow: synthetic feature vectors with survival times (left) are clustered through the network using our custom *PartialMultivariateLogrankloss* (center) to generate class predictions (right). **(b)** Probability density of the multivariate gaussian distributions used to sample the synthetic feature vectors from, color-coded by the three ground truth groups. **(c)** Weibull distributions used to sample survival times for each group from. **(d)** ROC curves showing performance of ground truth recovery with AUROC values of 0.96, 0.96, and 0.97 for classes 0, 1, and 2, respectively. **(e)** Precision-recall curves (PRC) showing performance of ground truth recovery with AUPRC values of 0.93, 0.94, and 0.93 for classes 0, 1, and 2, respectively. **(f)** Kaplan-Meier survival curves with 95% confidence intervals for the resulting three ground truth groups. **(g)** Kaplan-Meier survival curves with 95% confidence intervals for the three classes identified by the multilayer perceptron without prior knowledge of ground truth groupings.

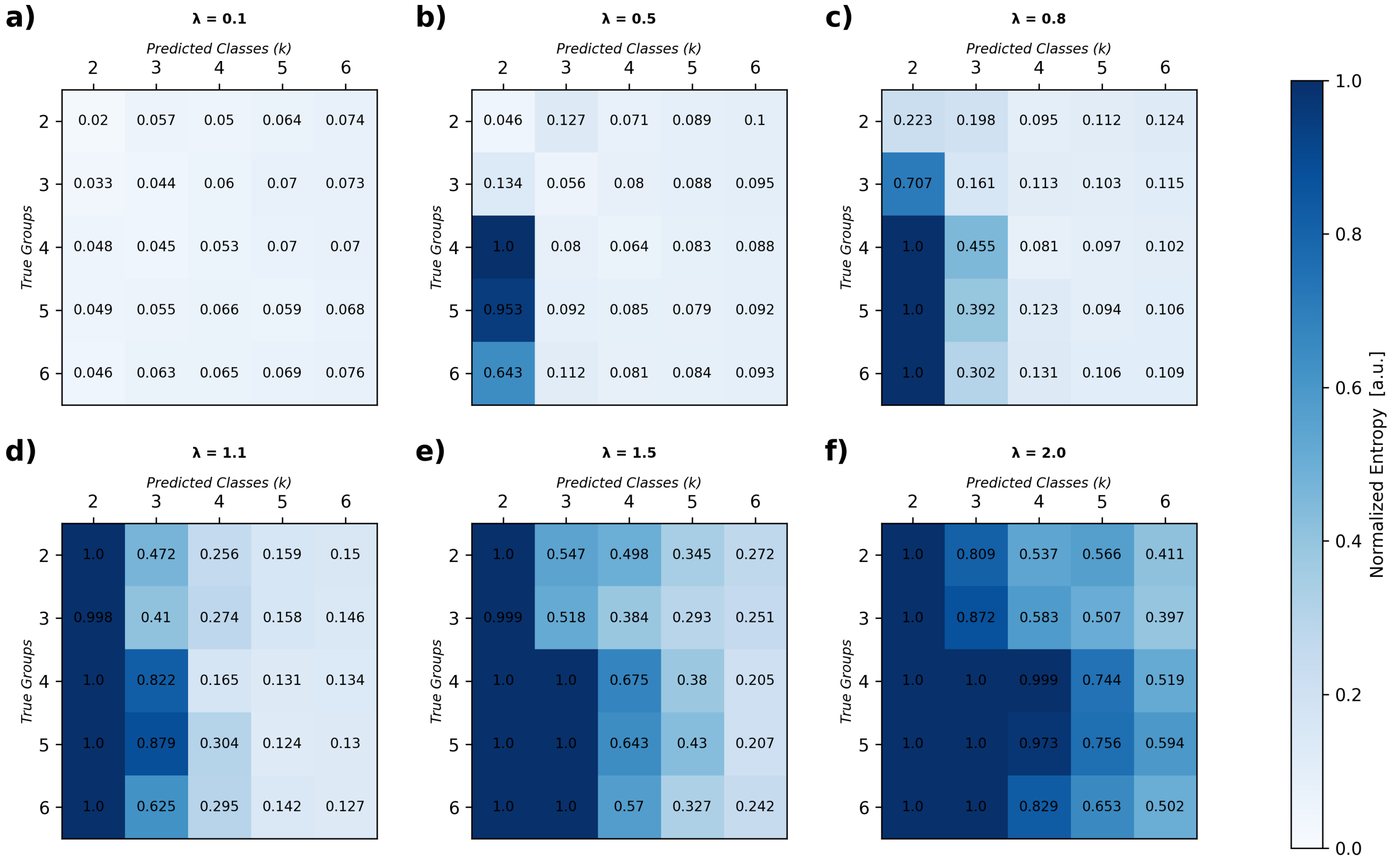

Supplementary Figure 2: **Simulation experiments evaluating varations of model paramter k, penalty weight λ and number of ground truth groups g.** Panels display heatmaps entropy in networks' predictions for combinations of g and k, evaluated across class-imbalance penalty weights a) λ=0.1, b) λ=0.5, c) λ=0.8, d) λ=1.1, e) λ=1.5 and f) λ=2.0. Entropy values are normalized by the maximum achievable entropy for the respective number of k and averaged over 10 independent simulations with different random seeds.

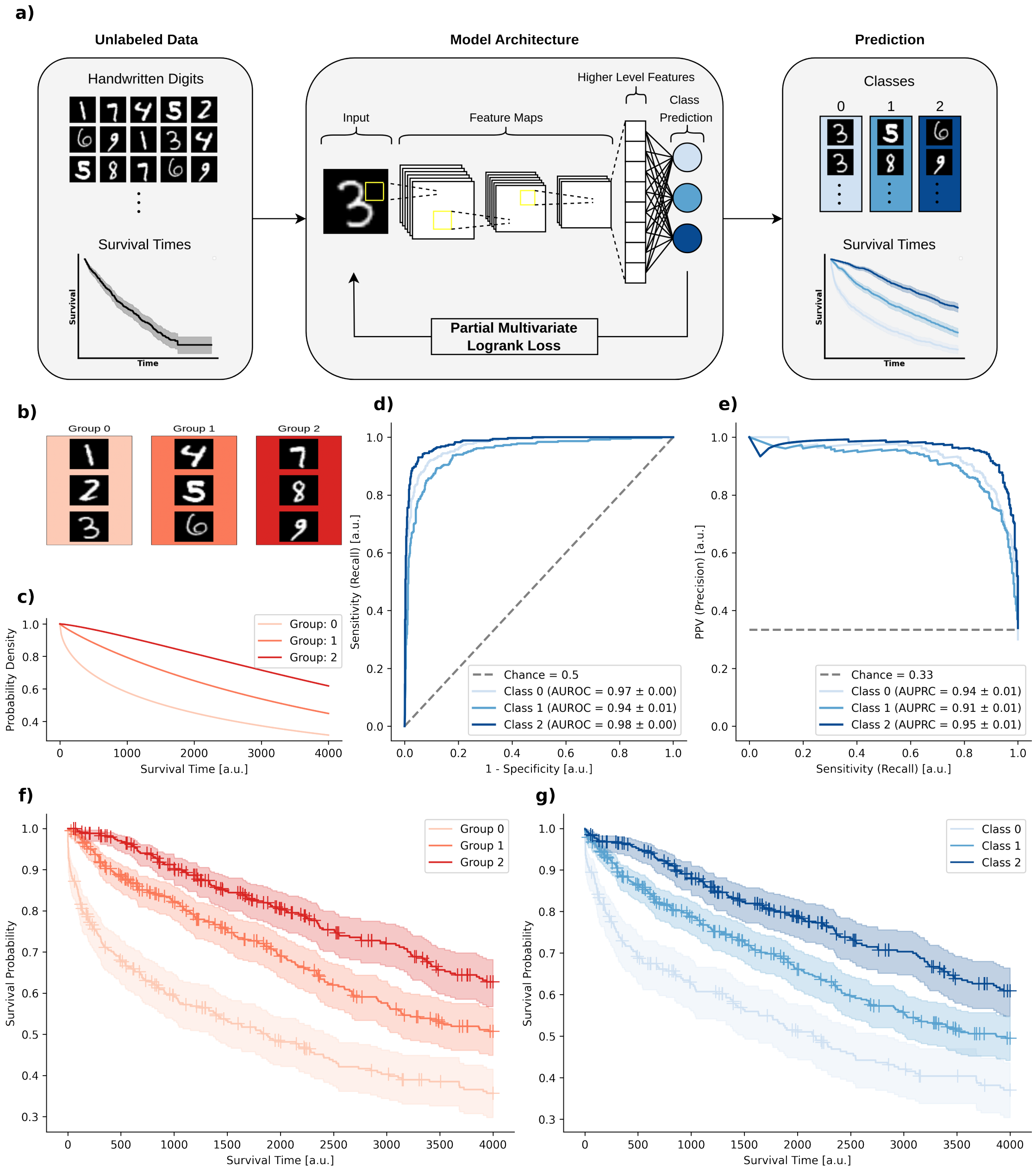

Supplementary Figure 3: **Stratification of handwritten digits according to their associated survival distributions.** **(a)** Architecture diagram showing the CNN workflow: MNIST handwritten digits with associated survival times (left) are processed through convolutional layers to extract features (center) and generate class predictions (right) using our custom *PartialMultivariateLogrankLoss*. **(b)** Representative MNIST digits from the three predefined groups: Group 0 (light red) contains digits 1-3, Group 1 (medium red) includes digits 4-6, and Group 2 (dark red) comprises digits 7-9. **(c)** Weibull distributions used to sample survival times for each digit group. **(d)** ROC curves showing discriminative performance of ground truth groupings with AUROC values of 0.97, 0.95, and 0.98 for Classes 0, 1, and 2, respectively. **(e)** Precision-recall curves (PRC) showing precision of models' predictions with AUPRC values of 0.93 0.91, and 0.96 for Classes 0, 1, and 2, respectively. **(f)** Kaplan-Meier survival curves with 95% confidence intervals for the resulting three ground truth groups. **(g)** Kaplan-Meier curves for the three classes identified by the CNN without prior knowledge of ground truth groupings.

Supplementary results of the Multiple Myeloma (MM) application

Biomarker distribution analysis

Analysis of biomarker distributions across the three identified clusters revealed clinically meaningful patterns that align with established disease biology (Figure 2c). The high-risk Cluster 0 exhibited significantly elevated levels of known adverse prognostic markers, including β-2-microglobulin (β2m) ($p < 0.001$), Creatinine (Cr) ($p < 0.001$), Lactate dehydrogenase (LDH) ($p < 0.001$), Calcium (Ca) ($p < 0.001$), White blood bells (WBC) ($p < 0.001$) and serum free light-chain (SFL) ($p < 0.001$), alongside reduced Albumin (Alb) ($p < 0.001$), M-Protein (M-Pr) ($p < 0.001$) and Hemoglobin (Hb) ($p < 0.001$) levels. Conversely, the favorable-risk Cluster 2 demonstrated lower β2m and higher Alb levels, consistent with established biomarkers indicating less advanced disease. All biomarkers showed statistically significant differences across the three clusters based on a Kruskal-Wallis test.

In the external validation setting, the biomarker distribution patterns across clusters remained consistent with the internal validation cohort (Figure 2f), with Cluster 0 showing elevated levels of the adverse prognostic β2m, LDH, and Cr, while displaying reduced Alb and Hb levels (all $p < 0.001$). The SHAP analysis in the external cohort (Figure 2g) further confirmed the reproducibility of feature importance rankings, with beforementioned markers maintaining their positions as the most influential parameters for risk classification.

Real-world translation of simulation experiments

Following our experiments in synthetic data (section Performance assessment of our method on simulated data; Supplementary Figure 4), we investigated the impact in variations of k and λ in the optimization of patient stratification in the CoMMpass dataset. Specifically, we investigated the impact distribution of the entropy and c-indices of our model's predicted class assignments across different configurations and compared its qualitative behaviors to those observed in our simulations For the different configurations, we trained models with penalty weights $\lambda \in \{0.001, 0.05, 0.1, 0.5, 2.0\}$ to stratify patients into $k \in \{2, 3, 4, 5, 6\}$ clusters.

Our analysis revealed a similar pattern with our synthetic data simulations. At lower values of λ, we observed that the predicted class assignments demonstrated a relatively uniform informativeness across different values for k, As the penalty weight increased, the entropy of predictions increased as well, while larger values for k allowed for higher informativeness in the presence of stronger penalties. Curiously, exploring the c-index across different combinations of k and λ suggested a "sweet spot" of optimal stratification, which appears to result from the best joint choice of k and λ, which can be found via hyperparameter search.

Notably, we did not conduct an analogous analysis on the Lung1 dataset due to the limited sample size of the dataset and the considerably increased complexity of the optimization problem, which precluded us from getting sensible fits for most configurations, which prevented us from exploring a parameter grid comparable to those used in our CoMMpass and synthetic data analyses.

Alignment with established clinical risk groups and treatment patterns

Finally, to test concordance with established clinical staging, we investigated alignment of the MM-MLP risk groups with established clinical risk groups (Supplementary Table 1). Here, we found a strong correlation between ISS and model-assigned group ($r_S = 0.74$, 95% CI: 0.71—0.77), which is expected because ISS components were among the features available to the model and which we previously showed were autonomously identified as major drivers of risk assignment. Interestingly, the correlation with the Revised International Staging System (R-ISS) was also moderately strong ($r_S = 0.61$, 95% CI: 0.56—0.65). Here, the strong correlation between ISS and R-ISS themselves ($r_S = 0.77$, 95% CI: 0.73—0.80) may presumably drive this alignment to a substantial degree, but nevertheless indicates that the model also aligns with the more biologically informed R-ISS.

Closer examination revealed that many R-ISS 2 patients were distributed into both model risk group 0 (high-risk) and group 2 (low-risk). We hypothesize that this pattern may reflect the interplay of two effects: (1) the high prior mass (approx. 60%) and heterogeneous outcomes for the intermediate-risk categories (a recognized challenge of the R-ISS[91]), and (2) the model's ability to refine integrate additional clinical signals (e.g. the previously identified markers of renal impairment and other features not included in clinical staging systems; see results section).

To address this hypothesis, we performed an an additional layer of analyses by expanding our shapley additive explanation (SHAP) analysis to examine feature contributions across all three risk groups (Supplementary Figure 5) more closely. Here, we found that the intermediary group exhibited combinations of both protective and adverse biomarker profiles within individual cases. For example, some patients displayed elevated β2m levels (typically associated with poor prognosis) alongside higher Alb levels (generally protective), while others showed the inverse pattern. This heterogeneous feature composition may explain the intermediate survival trajectory and demonstrates the existence of patients with

mixed-risk profiles that may require tailored therapeutic strategies distinct from those for clearly high- or low-risk patients. Thus, we hypothesize that, rather than contradicting the R-ISS, the MLP appears to recapitulate its determinants while providing additional discrimination within the intermediate group.

Lastly, therapy classes were evenly distributed across model groups, indicating that treatment heterogeneity was not a confounder of the learned risk groups (Supplementary Table 1). However, two exceptions are notable: a relatively higher proportion of bortezomib-based treatments in the high-risk group and a higher proportion of combined IMiD/carfilzomib regimens in the lowest-risk group. Albeit rather speculative, one possible interpretation for this would be that treating physicians preferentially selected bortezomib-containing regimens for patients with higher apparent disease burden or adverse clinical features (including renal dysfunction, where bortezomib is commonly favored), whereas lower-risk, fitter patients (who are more likely to proceed to SCT) received IMiD/carfilzomib combinations.

Taken together, these findings provide evidence that the MM-MLP aligns with the established clinical and biological risk groups ISS and R-ISS while refining intermediate cases through additional clinical signals. The modest associations treatment class are consistent with expected clinical decision patterns and do not suggest that treatment assignment may be a confounding factor for the model's risk stratification.

Transcriptomics model implementation and training

Lastly, to test the limits of our methodology, we sought to apply it to data characterized by very high dimensionality. Specifically, we used bulk mRNA sequencing data from bone marrow plasma cells comprising over 226,654 transcripts.

To construct a MLP for application to transcriptomic data in MM, we used the Salmon quantification output at the transcript level, generated with library type "unstranded", with expression values in TPM (Transcripts Per Million) from the MMRF CoMMpass study (Interim Analysis 2022). The sequencing dataset contained 226,654 transcripts from bone marrow plasma cells, which we used as input for the MLP to stratify patients into clusters with differential prognoses. The MLP was configured as follows:

$$\begin{aligned}&\mathit{Linear}\ 226654 \mapsto 256 + \mathit{ReLU}\\&\mathit{Linear}\ 64 \mapsto 64 + \mathit{ReLU}\\&\mathit{Linear}\ 64 \mapsto 10 + \mathit{Softmax}.\end{aligned}$$

The model was trained at a learning rate of $10^{-3.9}$, for 20 epochs with a mini-batch size of 128, using a uniform weight-decay of $10^{-2.7}$ applied to weights and biases. The class-imbalance penalty for our custom *PartialMultivariateLogRankLoss* was tuned to 10. The MM-MLP model architecture as well as the training hyperparameterization was obtained via Bayesian optimization using a Gaussian process sampler from optuna[29] (version 4.6.0). While the hyperparameter optimization yielded a stratification of patients into ten clusters, in the downstream analysis, we fused some of the intermediary clusters due to overlap in their survival trajectories, resulting to six final clusters.

Here, the model obtained using our method successfully stratified patients into six distinct prognostic clusters with decent discrimination ($c = 0.67 \pm 0.04$) and highly significant separation ($p < 10^{-16}$; Supplementary Figure 6a). Top ten transcripts driving assignment to both high-risk (Cluster 0) and low-risk (Cluster 5) groups, were investigated using SHAP. These determinants included transcripts that align with clinical expectations (e.g. immunoglobulin lambda variable 3-29 (IGLV3-29-201), Immunoglobulin kappa variable 1D-17 (IGKV1D-17-201), Cancer susceptibility 15 (CASC15-254), Fibroblast growth factor receptor 2 (FGFR2-210), etc.) as well as several novel, not yet characterized transcripts (Supplementary Figure 6b&c).

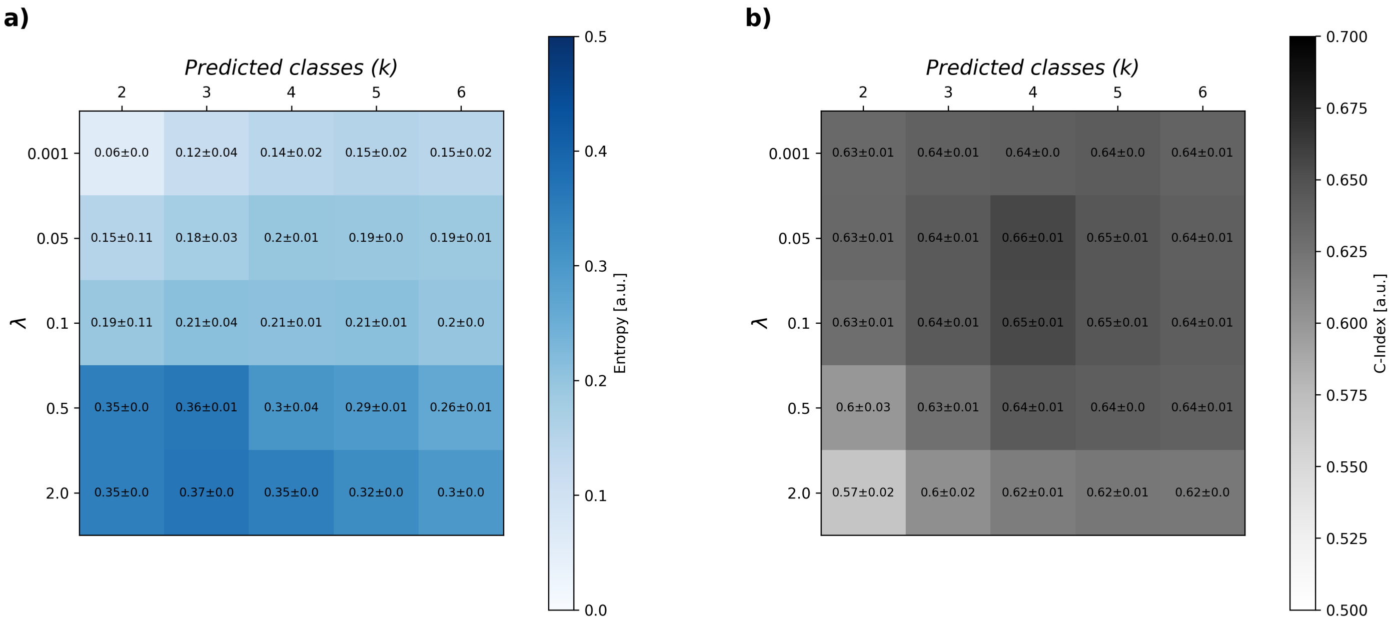

Supplementary Figure 4: **Entropy and C-Indices for varations of model paramter k and penalty weight λ for Myeloma-MLP. a)** Entropy in networks' predictions for combinations of predicted classes k and penalty weight λ. Entropy values are normalized by the maximum achievable entropy for the respective number of k. **b)** C-index from networks' predictions for combinations of predicted classes k and penalty weight λ. Values are reported as mean ± s.d. across models which were initialized using three different seeds. Metrics for each configuration were computed using 5-fold cross-validation and are thus based entirely on unseen patients.

Supplementary Table 1: Patient characteristics table for MM cohort separated by risk-group assigned by MM-MLP. r_S denotes the Spearman correlation coefficient between the respective characteristic and the MLP's risk-group assignment. Values are reported as median and 95% CI from a bootstrap analysis.

Characteristic	Total	Risk Group 0	Risk Group 1	Risk Group 2
**Gender**				
Female	294 (41%)	78 (39%)	75 (45%)	141 (39%)
Male	429 (59%)	123 (61%)	90 (55%)	216 (61%)
**Age**	63±11	66±11	64±11	62±10
**ISS** $r_S = 0.74$ (0.71—0.77)				
ISS 1	264 (37%)	2 (1%)	28 (17%)	234 (66%)
ISS 2	258 (36%)	43 (21%)	96 (58%)	119 (33%)
ISS 3	201 (28%)	156 (78%)	41 (25%)	4 (1%)
**R-ISS** $r_S = 0.61$ (0.56—0.65)				
R-ISS 1	132 (23%)	0 (0%)	10 (7%)	122 (47%)
R-ISS 2	348 (62%)	102 (60%)	107 (80%)	139 (53%)
R-ISS 3	85 (15%)	67 (40%)	17 (13%)	1 (0%)
**SCT** $r_S = 0.21$ (0.16—0.27)				
No	306 (42%)	121 (60%)	73 (44%)	112 (31%)
Yes	417 (58%)	80 (40%)	92 (56%)	245 (69%)
**Therapy Class**				
Bortezomib-based	140 (19%)	60 (30%)	43 (26%)	37 (10%)
Carfilzomib-based	4 (1%)	2 (1%)	1 (1%)	1 (0%)
Combined Bortezomib/Carfilzomib-based	1 (0%)			1 (0%)
Combined Bortezomib/IMIDs-based	449 (62%)	116 (58%)	92 (56%)	241 (68%)
Combined Bortezomib/IMIDs/Carfilzomib-based	34 (5%)	7 (3%)	8 (5%)	19 (5%)
Combined IMIDs/Carfilzomib-based	54 (7%)	4 (2%)	11 (7%)	39 (11%)
IMIDs-based	41 (6%)	12 (6%)	10 (6%)	19 (5%)

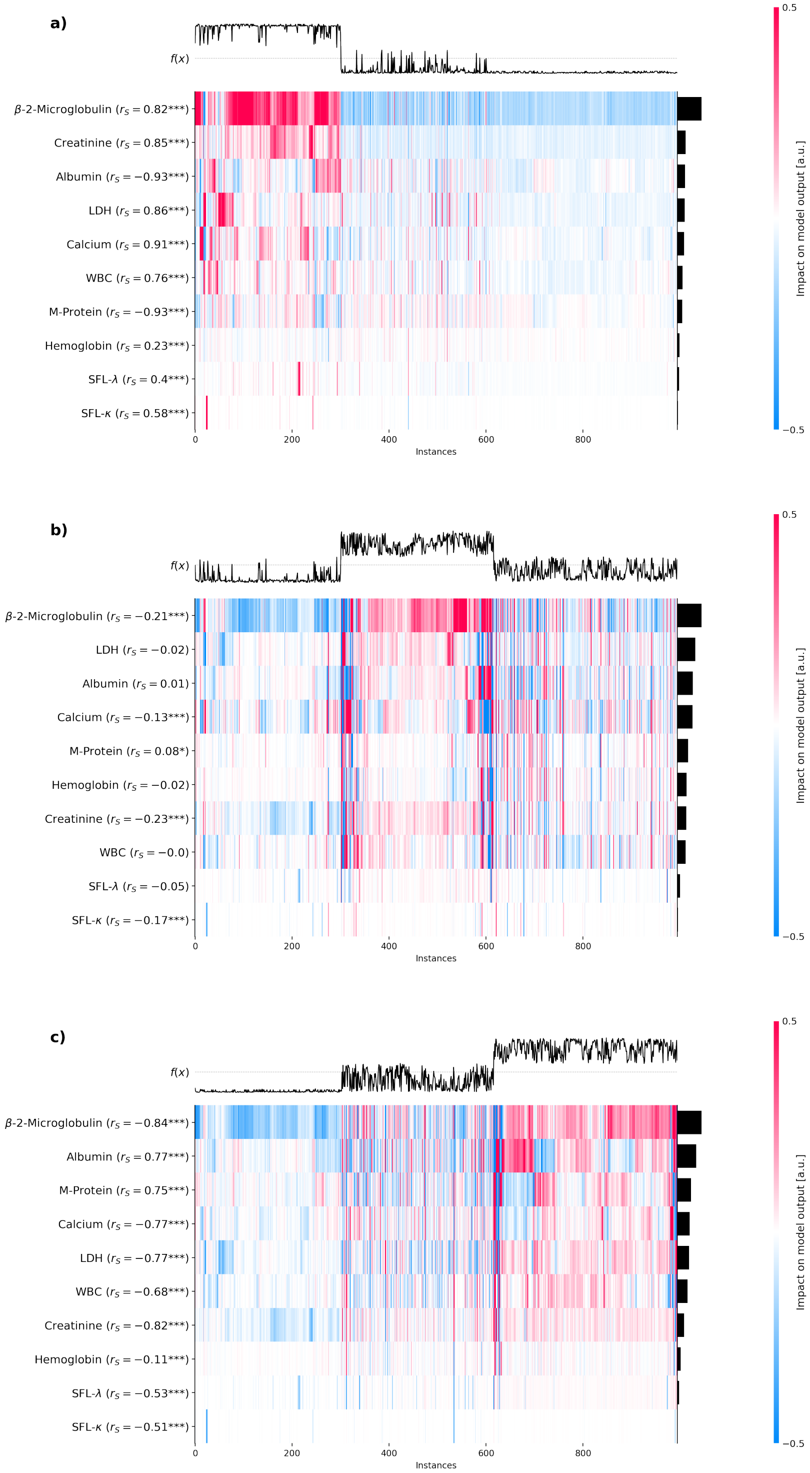

Supplementary Figure 5: **Patient level SHAP analysis revealing feature contributions for each risk cluster.** Panels (a), (b), and (c) display SHAP values for Clusters 0, 1, and 2, respectively, showing each feature's impact on the model's output for cluster assignment for each patient individually. Each column represents an individual patient, while rows show different blood parameters with their corresponding correlation coefficients (r_S) between feature values and SHAP values in parentheses. The color scale indicates the magnitude and direction of feature impact, with red representing positive contributions (higher values pushing toward cluster assignment) and blue representing negative contributions (lower values pushing away from cluster assignment). The f(x) plot at the top of each panel shows the approximated model output based on cumulative SHAP values across all features for each patient. Features are ordered by their overall importance for cluster discrimination, with the most influential parameters (β-2-Microglobulin, Creatinine, Albumin) appearing at the top.

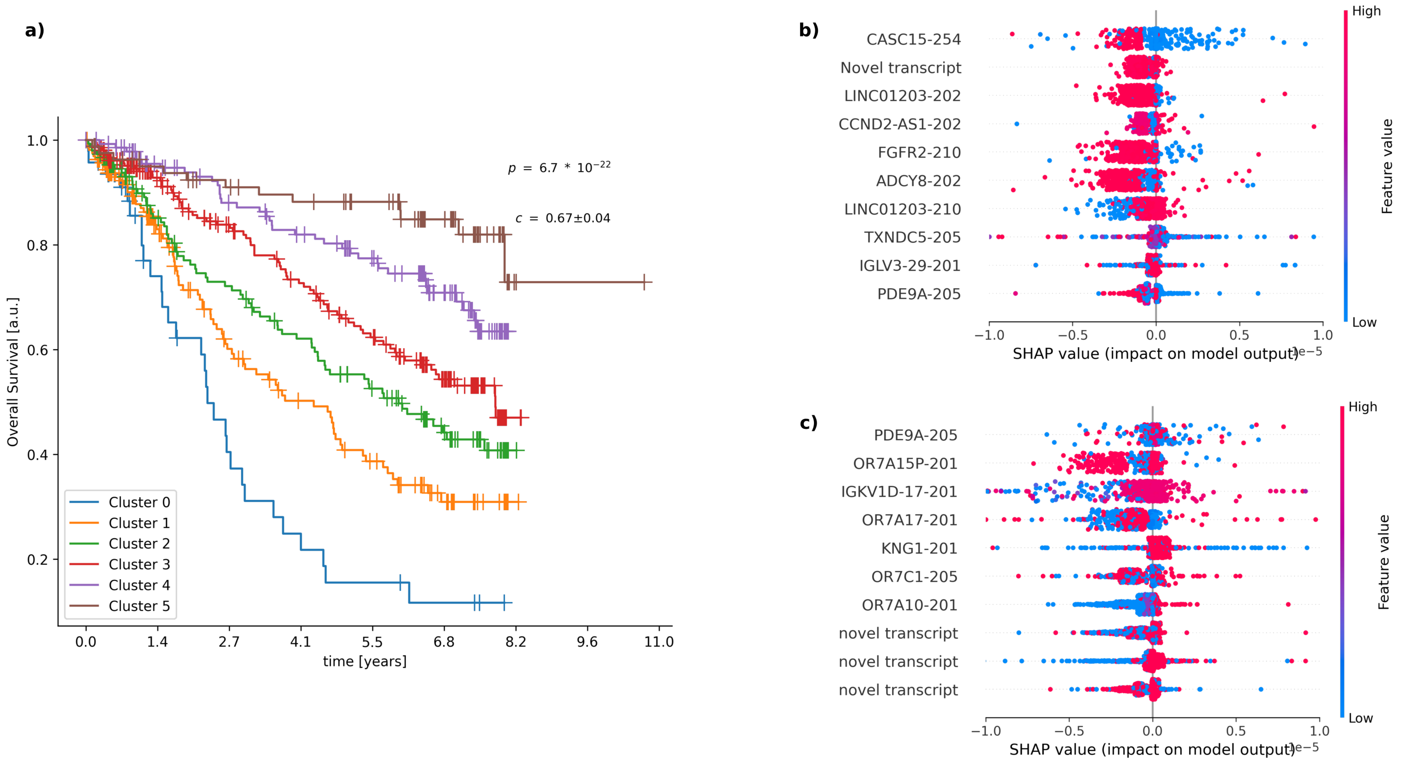

Supplementary Figure 6: **Molecular clustering of multiple myeloma patients based on bone marrow plasma cell transcriptomics.** **a)** Kaplan-Meier survival curves showing overall survival probability for six patient clusters identified by a MLP constructed using our method, based on bone marrow plasma cell mRNA sequencing data. Clusters demonstrate significant prognostic stratification ($p < 10^{-16}$), with Cluster 0 showing the poorest survival outcomes and Cluster 5 exhibiting the most favorable prognosis ($c = 0.67 \pm 0.04$). **b)** SHAP values for the top 10 transcripts influencing assignment to high-risk Cluster 0. **c)** SHAP values for the top 10 transcripts influencing assignment to low-risk Cluster 5. Red dots indicate high expression values while blue dots represent low expression values. The distribution of red (high expression) and blue (low expression) dots demonstrates how specific transcript expression patterns contribute to either prognosis classification. The model leverages 226,654 transcripts from bulk mRNA sequencing data to assign patients to prognostic clusters, hinting at molecular signatures associated with disease outcomes.

Supplementary Materials

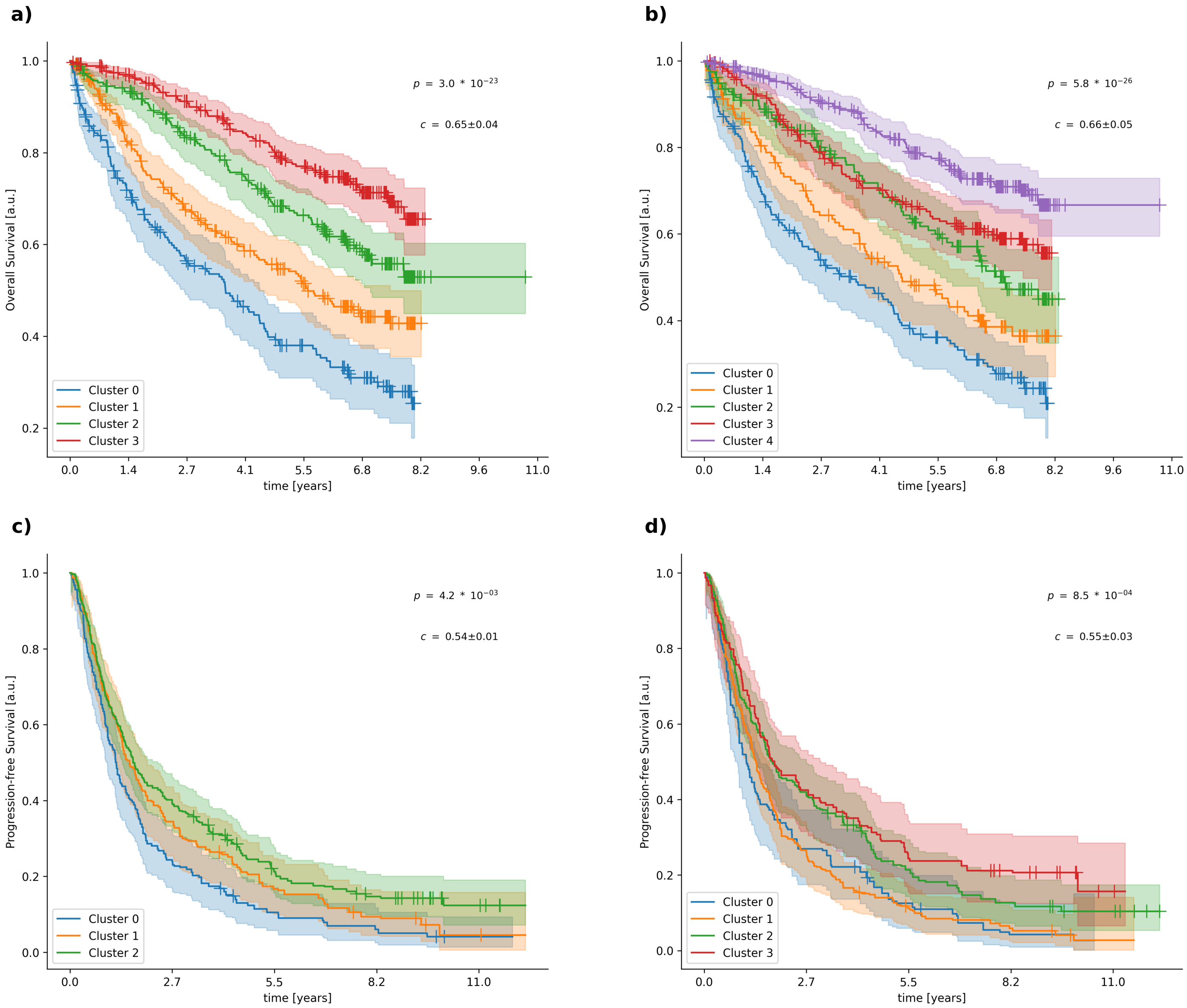

Supplementary Figure 7: **Kaplan-Meier survival curves demonstrating clustering-based risk stratification across different cancer types and cluster numbers.** Panels (a-b) show MM patient stratification analogous to our analyses in Figure Figure 2 with (a) k=4 clusters and (b) k=5 clusters, displaying distinct risk groups with significant survival differences. Panels (c-d) present Non-Small Cell Lung Carcinoma (NSCLC) patient stratification analogous to our analyses in Figure Figure 3 with (c) k=3 clusters and (d) k=4 clusters, also achieving statistically significant risk stratification. Each panel displays overall survival probability over time (years) for the respective number of risk clusters, with 95% confidence intervals shown as shaded regions. Cluster colors progress from blue (lowest risk) through orange, green, red, to purple (highest risk) where applicable. P-values are report statistical significance based on a multivariate logrank test. Concordance indices (c) are reported as mean±s.d. across cross-validation folds. All shown data were derived from the testing partitions of the 5 cross-validation-folds and are entirely based on unseen patients.

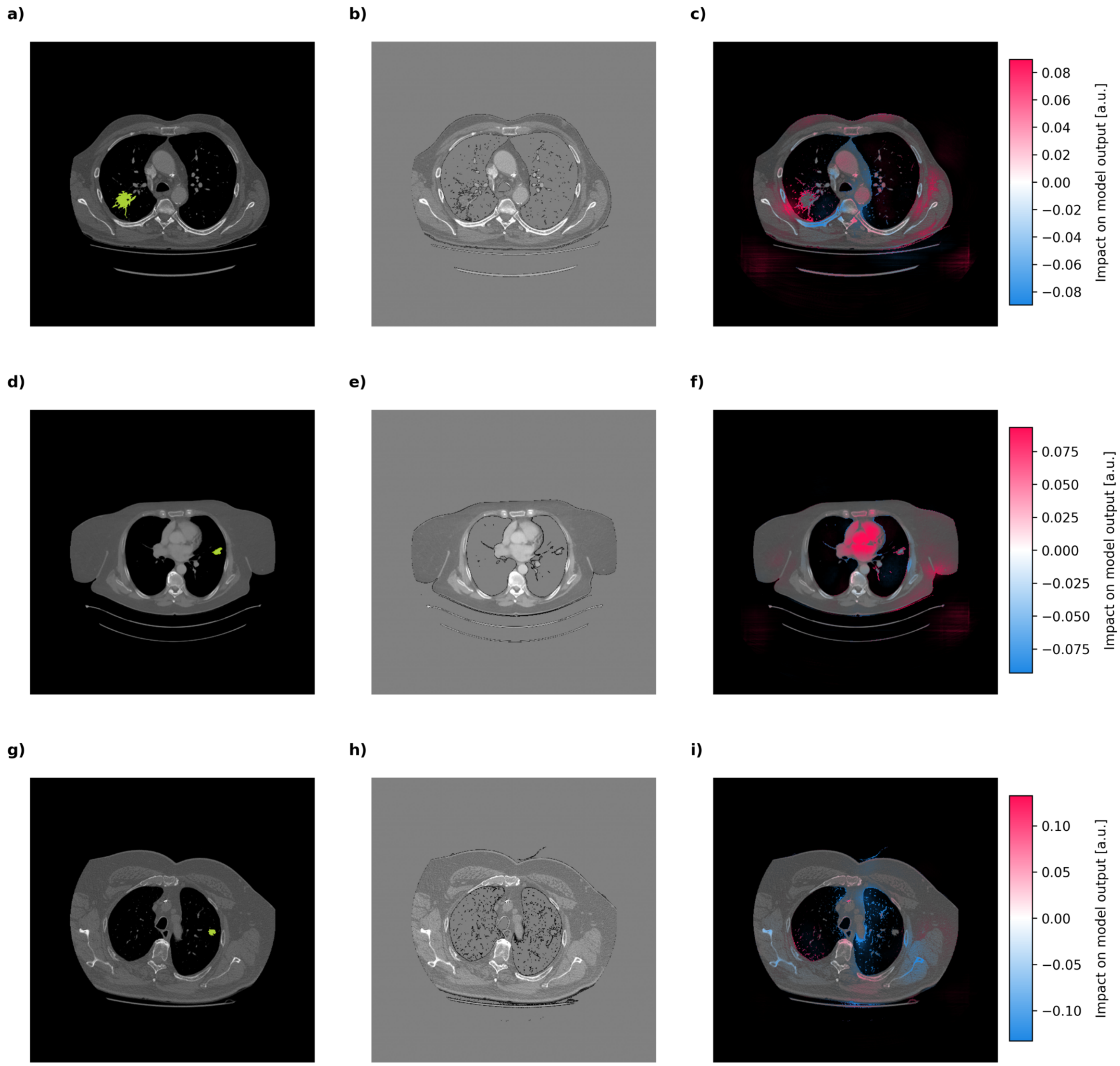

Supplementary Figure 8: **External application of CNN to Computed Tomography (CT) real-world clinical imaging data of Non-Small Cell Lung Carcinoma (NSCLC) patients treated of University Hospital Leipzig.** CT scan slices from three representative Non-Small Cell Lung Carcinoma (NSCLC) patients (rows a-c, d-f, and g-i) showing: manual tumor annotations by clinical experts highlighted in green (left column: a, d, g); reference CT images with zero-signal preserving contrast enhancement (middle column: b, e, h); and corresponding SHAP value heatmaps (right column: c, f, i) from the CNN trained on the Lung1 dataset. SHAP values represent pixel-level importance for the model's survival-based predictions. Color scale bars indicate SHAP value magnitude (arbitrary units), representing increased model attention in colored areas. All shown data were derived from real-world clinical imaging data acquired at our institution and are entirely based on unseen patients during training.
